# Acetophenone Mannich bases: study of ionic liquid catalysed synthesis and antioxidative potential of products

**DOI:** 10.1098/rsos.181232

**Published:** 2018-11-14

**Authors:** Vladimir P. Petrović, Dušica Simijonović, Vesna M. Milovanović, Zorica D. Petrović

**Affiliations:** Department of Chemistry, Faculty of Science, University of Kragujevac, Kragujevac 34000, Serbia

**Keywords:** ionic liquids, organocatalysis, spectral characterization, density functional theory, reaction mechanism, radical scavenging mechanisms

## Abstract

Three-component Mannich reaction of acetophenone or 4-iodoacetophenone with a variety of substituted anilines and benzaldehyde, catalysed with diethanolammonium chloroacetate, was performed under mild conditions. Mannich bases (**MBs**), of which five are new, were obtained in good to excellent yields. All compounds were characterized using elemental analysis, NMR and IR. In addition, detailed experimental and simulated UV–Vis spectral characterization of these compounds is presented here for the first time. *In vitro* antioxidative potential of synthetized **MBs** was evaluated using 2,2-diphenyl-1-picryl-hydrazyl radical and density functional theory (DFT) thermodynamical study. It was shown that compounds with anisidine moiety express moderate antioxidative activity. Mechanism of the organocatalysed Mannich reaction was thoroughly inspected by means of DFT. The reaction undergoes the hydrogen bonding-assisted mechanism. Moreover, the proposed rate determining step of the overall reaction is water elimination in the process of iminium ion formation. To the extent of our knowledge, this is the first detailed report on the influence of this type of catalyst on the formation of iminium ion, as a crucial intermediate for the whole reaction.

## 1. Introduction

The Mannich reaction is one of the most important methods for the formation of a new C–C bond and synthesis of aminocarbonyl molecules [1–4]. Thus obtained compounds can be versatile intermediates for the further synthesis of amino alcohols, amino acids, peptides, lactams and many other significant biomolecules.

Acetophenone-derived Mannich bases (**MBs**) are very interesting and important compounds in medicinal chemistry and drug discovery, because they act as important pharmacophores with promising activity [5–11]. It turned out that their cytotoxic activities against T-lymphocyte (Jurkat), renal carcinoma (Renca) and mammary (EMT6) carcinoma cells lines were significant [12,13]. In addition, these compounds possess good antimicrobial [14–17], as well as anti-inflammatory activities [18]. **MBs** are a wide, inexhaustible and still unexplored topic. Therefore, further investigations should be directed towards the synthesis of new compounds of this type.

The classical Mannich reactions of aldehydes, ketones and amines can be catalysed with Lewis acids and bases [19–21], Brønsted acids [22,23], rare metal salt [24,25] and organocatalysts [26–28]. However, these catalysts are accompanied by different disadvantages, such as harsh reaction condition, long reaction times or difficult separation of the reaction product, etc., which limit their usage. Recently, ionic liquids (ILs), also known as liquid salts, were used as a promising alternative to the conventional catalysts [9,29–32]. Owing to their excellent properties, such as low vapour pressure, reusability and high thermal and chemical stability, ILs are successfully applied to a wide range of reactions, either as catalysts or solvents, or dual catalyst–solvent. The usage of Brønsted-acidic ILs is in increase due to their high efficiency, relatively low prices, recycling performance and due to the easy separation of products and catalyst from the reaction mixture.

So far, mechanistic studies have been carried out for different catalysts, including proline, Brønsted and Lewis acids, and a variety of different organocatalysts [8,33–41]. However, most of them deal with the part of the reaction where new C–C bond is being established, while the first step, formation of the iminium ion is usually omitted and taken for granted.

In this study, we report the three-component Mannich reaction of acetophenones with a variety of aromatic amines and benzaldehyde, catalysed with diethanolammonium chloroacetate ([HDEA][ClAc]) IL (liquid salt). To our knowledge, the literature is missing data on detailed mechanistic studies for the formation of iminium ion catalysed with this type of catalyst, as well as the whole mechanistic studies of acetophenone-derived **MBs**. Therefore, one of our goals was to examine the mechanism of the reaction in order to distinguish the role of the used catalyst in the formation of the iminium ion, as well as its role in the subsequent C–C coupling step of the reaction. Examination of the influence of the explicit solvent molecules on this reaction was investigated, also. For this purpose, we used density functional theory (DFT). In addition, our aim was to structurally characterize the obtained products using NMR and UV–Vis spectroscopies. UV–Vis spectra were simulated using TD-DFT and compared to the experimental one. Obtained compounds were examined for their antioxidative potential experimentally and theoretically.

## 2. Material and methods

### 2.1. Experimental set-up

Chloroacetic acid, benzaldehyde, aniline, 4-chloroaniline, 4-fluoroaniline, *p*-toluidine, *p*-anisidine, acetophenone, 4-iodoacetophenone and 1,1-diphenyl-2-picryl-hydrazyl were obtained from Aldrich Chemical Co. Diethanolamine (DEA) was purchased from Fluka. All common chemicals were of reagent grade.

The ^1^H NMR and ^13^C NMR spectra were run in CDCl_3_ on a Varian Gemini 200 and 50 MHz spectrometer. The UV–Vis spectra were measured at room temperature within the 200–500 nm range on the Agilent Technologies, Cary 300 Series UV–Vis Spectrophotometer. A solution of 2.5 × 10^−5^ M of each compound was prepared in methanol and then 2 ml of the corresponding solution was injected into the 10 mm quartz cell and the spectrum was recorded. The IR spectra in the solid state were recorded on a PerkinElmer Spectrum One FT-IR spectrometer using KBr pellet technique. The resolution of the scanning was 4.0 cm^−1^ at 16 scans. Melting points were determined on a Mel-Temp capillary melting points apparatus, model 1001, and obtained values are compared to those available in the literature for known compounds [42–44]. Elemental microanalyses for carbon, hydrogen and nitrogen were performed at the Faculty of Chemistry, University of Belgrade.

### 2.2. General procedure for the synthesis of Mannich bases

Benzaldehyde (1 mmol), amine (aniline, 4-chloroaniline, 4-fluoroaniline, *p*-toluidine or *p*-anisidine) (1 mmol), aromatic ketone (acetophenone or 4-iodoacetophenone (1.2 mmol), ionic liquid—diethanolammonium chloroacetate [HDEA][ClAc] as catalyst (20 mol%) and 1 ml of ethanol were placed in flask and stirred at room temperature for 24 h. When the reaction was completed, the solid product was separated by filtration and washed with ethanol. The **MBs** were obtained by recrystallization from chloroform and propanol 2 : 1 (v/v) and identified by ^1^H NMR, ^13^C NMR, IR spectroscopy, melting point and elemental analysis. Upon the evaporation of the solvent, the remaining catalyst was used in new experiments. It is worth highlighting that there was no significant loss in the recycled catalyst activity, i.e. the yields were up to 5% lower. The characterizations of the new compounds are given in main part of the manuscript, while for known compounds in the electronic supplementary material, as well as ^1^H and ^13^C NMR spectra for all compounds.

3-(4-Chlorophenylamino)-1-(4-iodophenyl)-3-phenylpropan-1-one (**MB1**): white powder; ^1^H NMR (200 MHz, CDCl_3_): *δ* = 3.39 (dd, *J* = 6.3, 1.9 Hz, 1H), 3.41 (d, *J* = 1.1 Hz, 1H), 4.57 (br s, 1H), 4.93 (t, *J* = 6.3 Hz, 1H), 6.48 (d, *J* = 2.1 Hz, 1H), 6.45 (d, *J* = 2.1 Hz, 1H), 7.04 (d, *J* = 2.0 Hz, 1H), 7.00 (d, *J* = 2.1 Hz, 1H), 7.43–7.21 (m, 5H), 7.55 (d, *J* = 1.8 Hz, 1H), 7.59 (d, *J* = 1.9 Hz, 1H), 7.78 (d, *J* = 1.8 Hz, 1H), 7.81 (d, *J* = 1.8 Hz, 1H); ^13^C NMR (50 MHz, CDCl_3_): *δ* = 45.99, 54.89, 54.94, 101.50, 101.59, 115.02, 122.66, 126.26, 127.60, 128.96, 129.48, 136.00, 138.05, 142.28, 145.47, 197.45; IR (cm^−1^): 3383, 1673, 1579, 1501, 1286; UV (*λ*_max_) nm: 202, 260.5, 314 nm; C_21_H_17_ClINO (FW = 461.73): C, 54.63; N, 3.03; H, 3.71%; found: C, 54.83; N, 3.02; H, 3.72%.

1-(4-Iodophenyl)-3-phenyl-3-(phenylamino)propan-1-one (**MB2**): white crystals; ^1^H NMR (200 MHz, CDCl_3_): *δ* = 3.42 (dd, *J* = 6.4, 2.5 Hz, 2H), 4.51 (br s, 1H), 5.03–4.94 (m, 1H), 6.60–6.51 (m, 2H), 6.72–6.61 (m, 1H), 7.09 (dd, *J* = 8.5, 7.4 Hz, 2H), 7.37–7.21 (m, 5H), 7.45–7.37 (m, 2H), 7.59 (d, *J* = 8.7 Hz, 2H), 7.80 (d, *J* = 8.7 Hz, 2H); ^13^C NMR (50 MHz, CDCl_3_): *δ* = 46.7, 54.8, 101.36, 113.88, 117.94, 126.34, 127.43, 128.83, 129.12, 129.50, 136.09, 138.00, 142.74, 146.88, 197.54; IR (cm^−1^): 3378, 1669, 1578, 1508, 1288; UV (*λ*_max_) nm: 202, 259 nm; C_21_H_18_INO (FW = 427.29): C, 59.03; N, 3.28; H, 4.25%; found: C, 59.24; N, 3.27; H, 4.26%.

3-((4-Fluorophenyl)amino)-1-(4-iodophenyl)-3-phenylpropan-1-one (**MB3**): white crystals; ^1^H NMR (200 MHz, CDCl_3_): *δ* = 3.39 (dd, *J* = 6.3, 1.8 Hz, 2H), 4.41 (br s, 1H), 4.90 (t, *J* = 6.3 Hz, 1H), 6.55–6.42 (m, 2H), 6.85–6.71 (m, 2H), 7.45–7.21 (m, 5H), 7.56 (d, *J* = 1.9 Hz, 1H), 7.59 (d, *J* = 1.9 Hz, 1H), 7.78 (d, *J* = 1.9 Hz, 1H), 7.81 (d, *J* = 1.8 Hz, 1H); ^13^C NMR (50 MHz, CDCl_3_): *δ* = 46.17, 55.41, 101.52, 114.83, 114.98, 115.32, 115.76, 126.31, 127.53, 128.90, 129.49, 135.97, 138.02, 142.59, 143.26; IR (cm^−1^): 3370, 1668, 1576, 1509, 1283; UV (*λ*_max_) nm: 202, 264.5 nm; C_21_H_17_FINO (FW = 445.28): C, 56.65; N, 3.15; H, 3.85%; found: C, 56.45; N, 3.16; H, 3.86%.

1-(4-iodophenyl)-3-phenyl-3-(p-tolylamino)propan-1-one (**MB9**): white powder; ^1^H NMR (200 MHz, CDCl_3_): *δ* = 2.18 (s, 3H), 3.40 (dd, *J* = 6.4, 2.7 Hz, 2H), 4.95 (t, *J* = 0.8 Hz, 1H), 6.55–6.41 (m, 2H), 6.90 (d, *J* = 8.2 Hz, 2H), 7.50–7.17 (m, 5H), 7.66–7.51 (m, 2H), 7.87–7.73 (m, 2H), ^13^C NMR (50 MHz, CDCl_3_): *δ* = 20.32, 46.14, 55.02, 114.02, 126.34, 127.13, 127.37, 128.81, 129.51, 129.61, 137.97, 142.88, 144.54, 197.62; IR (cm^−1^): 3384.37, 1676.44, 1580.73, 1521.06, 1289.64; UV (*λ*_max_) nm: 202.5, 259.5, 329 nm; C_22_H_20_INO (FW = 441.31): C, 59.88; N, 3.17; H, 4.57%; found: C, 59.67; N, 3.18; H, 4.59%.

1-(4-iodophenyl)-3-((4-methoxyphenyl)amino)-3-phenylpropan-1-one (**MB10**): white powder ^1^H NMR (200 MHz, CDCl_3_): *δ* = 3.39 (dd, *J* = 6.4, 1.4 Hz, 2H), 3.69 (s, 2H), 4.24 (br, s, 1H), 4.90 (t, *J* = 6.4 Hz, 1H), 6.60–6.45 (m, 2H), 6.76–6.60 (m, 2H), 7.25 (d, *J* = 8.6 Hz, 1H), 7.41 (d, *J* = 6.7 Hz, 3H), 7.59 (d, *J* = 8.5 Hz, 2H), 7.76–7.90 (m, 3H), ^13^C NMR (50 MHz, CDCl_3_): *δ* = 46.23, 55.70, 101.35, 114.77, 115.45, 126.40, 127.40, 128.81, 129.51, 136.09, 137.99, 141.04, 142.98, 152.52, 197.68; IR (cm^−1^): 3381.94, 1673.46, 1580.36, 1513.35, 1263.92; UV (*λ*_max_) nm: 201.5, 269.5, 314 nm; C_22_H_20_INO_2_ (FW = 457.31): C, 57.78; N, 3.06; H, 4.41%; found: C, 57.58; N, 3.07; H, 4.42%.

#### 2.2.1. DPPH free radical scavenging assay

The free radical scavenging activity of the examined compounds was performed using the DPPH method [45]. DPPH solution (1 ml, 0.05 mM) in methanol was mixed with the tested compound (20 µl of compound solution in DMSO and 980 µl of methanol). After incubation at room temperature for 20 and 60 min, the absorbance was determined spectrophotometrically at 517 nm. Methanol was used as a control solution. IC_50_ values represent the concentration necessary to obtain 50% of a maximum scavenging capacity. Nordihydroguaiaretic acid (NDGA) was used as positive control. All measurements were performed on three replicates. The results are presented as mean values ± standard deviation (s.d.) of three independent measurements (electronic supplementary material, tables S4 and S5).

### 2.3. Details of calculations

All calculations were performed with the Gaussian 09 software [46]. M052X functional in conjunction with 6–311++G(d,p) basis set for all atoms except iodine, where 6–311G(d,p) was used [47,48]. The geometrical parameters of all stationary points were optimized in ethanol (*ɛ* = 24.3) using the conductor-like solvation model (CPCM) [49,50]. The nature of all calculated structures was determined by frequency calculations: all positive eigenvalues for equilibrium structures, and one negative eigenvalue for transition states. The natural bond orbital analysis (Gaussian NBO version) was performed for all structures. Simulations of UV–Vis spectra were performed using TD-DFT and structures optimized in methanol, because experimental spectra were acquired using this solvent. For the simulation of UV–Vis spectra M052X and B3LYP methods were used. The thermodynamic parameters and reaction enthalpies were obtained by the optimization of all relevant species in methanol, because it was used for experimental *in vitro* radical scavenging determination, using M052X method, 6–311++G(d,p) basis set for all atoms except iodine, where 6–311G(d,p) or def2-TZVP (Triple-Zeta-Valence basis set with polarization and effective core potentials) basis sets were used, and the CPCM [51].

## 3. Results and discussion

**Scheme 1.**
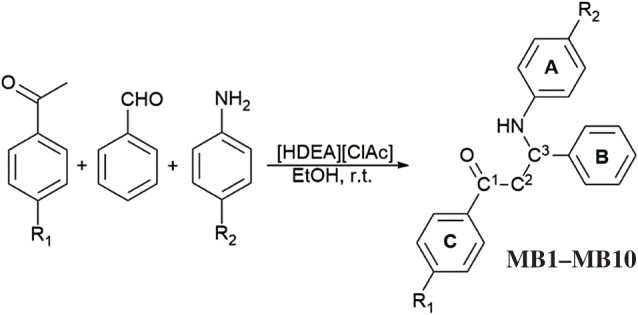
General reaction of acetophenones with benzaldehyde and some anilines. Description of the substituents *R*_1_ and *R*_2_ is given in table 2.

In an extension of our study of the Mannich coupling reaction, we conducted reactions of benzaldehyde, anilines and aromatic ketones (acetophenone or 4-iodoacetophenone), in the presence of catalytic amount of [HDEA][ClAc]. To optimize the reaction conditions, the reaction of aniline, benzaldehyde and acetophenone was performed using different amounts of [HDEA][ClAc] and diethanolammonium acetate [HDEA][Ac] as catalysts at room temperature (table 1). Ethanol was used as a solvent. Initially, in the presence of 10 and 15 mol% of catalysts, the product was obtained in moderate yields with both catalysts. When reactions were carried out in the presence of 20 mol% of the catalysts, the yields were notably better. Further increase in the catalyst amount to 25 mol% did not affect the progress of the reaction yield. However, reactions catalysed with [HDEA][ClAc] provided higher yields. To test whether the reaction proceeds without a catalyst, the reaction was performed in ethanol, but without [HDEA][ClAc] or [HDEA][Ac]. After 24 h, the Schiff base (**SB**) was the only product. Based on the obtained results, [HDEA][ClAc] was selected as a catalyst for further reactions. It was shown that catalytic amount of 20 mol% [HDEA][ClAc] and a small amount of ethanol as solvent (1 ml), at room temperature for 24 h, provided optimal reaction conditions. Under these conditions, reactions of benzaldehyde with different substituted anilines and with acetophenone or 4-iodoacetophenone were performed (scheme 1 and table 2). This way, all **MBs** (**MB1**–**MB10**) were obtained in moderate to good yields (75–90%). The products **MB1**–**MB3**, **MB9** and **MB10** obtained in reactions with 4-iodoacetophenone are newly synthesized compounds.

**Table 1.** Screening of catalytic efficiency of ILs. Reaction conditions: aniline (1 mmol), benzaldehyde (1 mmol), acetophenone (1.2 mmol); solvent: ethanol (1 ml); temperature: room temperature; n.r.: no reaction.

entry	amount of catalyst	yield %
1	[HDEA][ClAc]/[HDEA][Ac] 10 mol%	76/65
2	[HDEA][ClAc]/[HDEA][Ac] 15 mol%	82/74
3	[HDEA][ClAc]/[HDEA][Ac] 20 mol%	91/80
4	[HDEA][ClAc]/[HDEA][Ac] 25 mol%	91/82
5	none	n.r.

**Table 2.** Mannich reaction of anilines, benzaldehyde and acetophenones (molar ratios 1 : 1 : 1.2) in the presence of 20 mol% [HDEA][ClAc] as catalyst.

**MB**	*R*_1_	*R*_2_	isolated yield (%)	m.p. (°C)	m.p. from ref (°C)
**MB1**	I	Cl	90	159–160	—
**MB2**	I	H	88	161–163	—
**MB3**	I	F	84	156–157	—
**MB4**	H	H	91	167–169	168–170 [20,42,43]
**MB5**	H	Cl	91	167–168	169–171 [20,42–44]
**MB6**	H	F	87	164–166	163–164 [42]
**MB7**	H	CH_3_	85	167–168	169–171 [20,42–44]
**MB8**	H	OCH_3_	78	168–169	166–169 [42,44]
**MB9**	I	CH_3_	82	160–161	—
**MB10**	I	OCH_3_	75	150–151	—
without catalyst	H	H	n.r.	—	—

It is important to emphasize that, under these optimal conditions, only formation of the Mannich product was observed, unlike other similar Mannich reactions where aldols were detected as side products [52]. Reactions were carried out with benzaldehyde and differently substituted anilines. The highest yields were obtained in cases where in the para position of aniline are electron withdrawing halogen substituents or with the aniline itself (table 2).

### 3.1. ^1^H NMR and UV–Vis spectral characterization of **MBs**

All obtained products were characterized by NMR, IR and UV–Vis spectra, elemental analysis and melting points. It is worth pointing out that UV–Vis spectral characterization of these compounds will be discussed here for the first time. The signals observed in the ^1^H NMR spectra of the **MBs** are given in table 3, and corresponding atoms labelling in scheme 1. In the ^1^H NMR spectra of all **MBs**, detected signals originating from C^2^ methylene group appear in the region 3.30–3.59 ppm as doublet of doublet or multiplets. Proton bonded for asymmetric C^3^ carbon atom appears in the range of 4.89–5.03 ppm as triplet or multiplets. In the spectra of the **MBs** protons of the amino group appeared at about 4.50 ppm as broad singlets exchangeable with D_2_O. The protons attached to the aromatic carbons from ring **A**, **B** and **C** resonated as multiplets and doublets in the range of 6.41–7.09, 7.16–7.59 and 7.37–7.98 ppm, respectively.

**Table 3.** Chemical shifts of protons (ppm) in the **MBs** skeleton of **MB1–MB10** (^1^HNMR spectra).

	C^2^-H	C^3^-H	ArH		
			**A**	**B**	**C**
**MB1**	3.39	4.93	6.48–7.00	7.21–7.43	7.55–7.81
**MB2**	3.42	4.94–5.03	6.51–7.09	7.21–7.37	7.37–7.80
**MB3**	3.39	4.90	6.42–6.71	7.21–7.45	7.56–7.81
**MB4**	3.32–3.59	5.00	6.55–7.08	7.21–7.47	7.39–7.90
**MB5**	3.33–3.56	4.94	6.47–7.02	7.16–7.48	7.39–7.92
**MB6**	3.30–3.57	4.92	6.39–6.78	7.27–7.44	7.48–7.91
**MB7**	3.45	4.94–5.00	6.48–6.89	7.14–7.43	7.45–8.03
**MB8**	3.35–3.54	4.89–4.95	6.58–6.73	7.17–7.40	7.41–7.98
**MB9**	3.40	4.95	6.41–6.90	7.17–7.50	7.51–7.87
**MB10**	3.39	4.90	6.45–6.76	7.25–7.59	7.59–7.90

**Figure 1.**
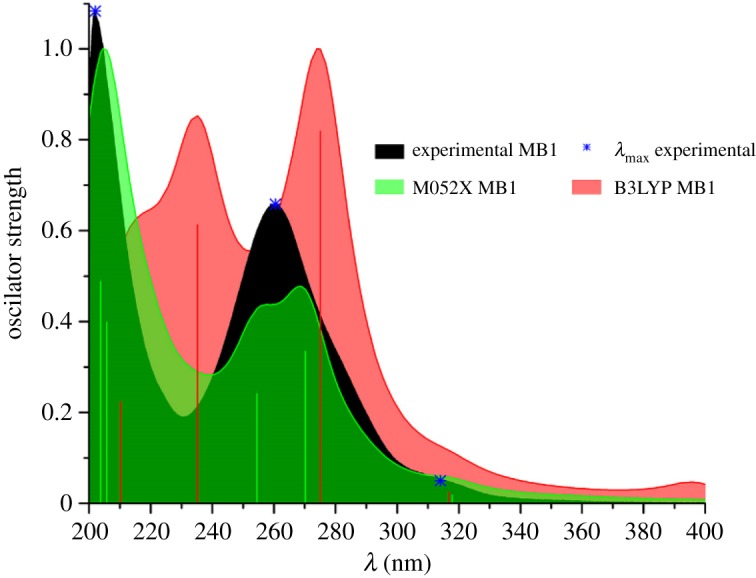
Experimental and simulated UV–Vis spectra of **MB1**. Simulated spectra are plotted using Lorentzian line shape and half-width 10.

In all experimental UV–Vis spectra of **MB1**–**MB10**, there are two major absorption bands around 205 and 260 nm, as well as a small band around 310 nm (figure 1 and electronic supplementary material, figure S3). To explore which transitions are responsible for the appearance of each absorption band, UV–Vis spectra of these compounds were simulated using TD-DFT (figure 1 and electronic supplementary material, figure S3), and Kohn–Sham orbitals were constructed (electronic supplementary material, figures S4–S13), while electron transitions and corresponding orbital energies are provided in electronic supplementary material, tables S1–S4. Spectra were simulated using M052X and B3LYP methods. Surprisingly, the B3LYP method failed in the reproduction of absorption spectra. Namely, in all spectra bands are either significantly redshifted or completely absent. Fleming *et al*. showed that the inaccuracy in *λ*_max_ can be caused by the solvation model used [53]. On the other hand, in their study, all experimental bands were present in simulated spectra. Bearing in mind that in our study some of the bands were completely absent when the B3LYP method was used, results obtained with the M052X method will be discussed here.

In all M052X simulated spectra, absorption bands are significantly redshifted. For this reason, simulated values were scaled using scaling factor obtained with the least square method. Here, details on the origin of absorption bands of **MB1** will be presented, while all other relevant data regarding compounds **MB2**–**MB10** is provided in the electronic supplementary material. Experimental absorption band around 202 nm is a consequence of several electronic transitions, represented with two bands found at 204 and 206 nm. The first one is a consequence of HOMO-5 to LUMO + 1 electron transition, while the second one of HOMO-4 to LUMO + 2, HOMO-4 to LUMO + 4, HOMO-3 to LUMO + 3 electron transitions. Based on the localization of orbitals responsible for these electronic transitions, as well as on their relative energies (electronic supplementary material, figure S4, tables S1 and S3) one can see that they are a consequence of large energetical but small spatial separation. Simulated spectra revealed that experimental band at 260 nm is also a consequence of several electronic transitions, appearing as two bands at 235.2 and 275 nm. Lower wavelength band is a consequence of relatively small spatial separation (HOMO to LUMO + 4, HOMO to LUMO + 5), while the higher wavelength band of relatively smaller energy gap (HOMO-2 to LUMO). Small absorption band at 314 nm appears owing to HOMO to LUMO electron transition, represented by the smallest energy gap, but also with a relatively small spatial gap.

### 3.2. Radical scavenging activity of **MBs**

*In vitro* antioxidant activity of synthetized **MBs** was determined on the basis of their interaction with the 1,1-diphenyl-2-picryl-hydrazyl (DPPH) stable free radical (table 4 and electronic supplementary material, table S5). It was found that, except for anisidine **MB8** and **MB10** bases, all investigated compounds are poor radical scavengers. **MB8** and **MB10** exhibited the best, but still moderate scavenging activities, with IC_50_ values of 48.4 and 109.3 µM (table 4). Since all investigated compounds do not have phenolic OH group, it is reasonable to expect that the NH group is responsible for the scavenging of DPPH radical. Furthermore, it is obvious that the presence of the methoxy group in the para position of the ring **A** plays a decisive role in the antioxidative activity. Namely, only presence of this strong electron donating group in the aniline moiety of **MBs** induced increased antiradical activity.

**Table 4.** Experimental IC_50_ (µM) values for inactivation of DPPH free radical, calculated thermodynamical parameters (kJ mol^−1^) of antioxidant mechanisms for **MB-8** and **MB-10** and reaction enthalpies (kJ mol^−1^) for the reactions of these compounds with the selected radicals in methanol.

	HAT	SET-PT		SPLET		HAT	SET-PT		SPLET	
	**MB-8**					**MB-10**				
IC_50_ (µM)	48.4 ± 0.1^a^					109.3 ± 0.3^a^				

thermodynamical parameters (kJ mol^−1^)										
property	BDE	IP	PDE	PA	ETE	BDE	IP	PDE	PA	ETE
	354	435	81	235	281	354	433	82	231	284
	343	407	97	243	261	343	408	96	242	263

reaction enthalpies (kJ mol^−1^)										
radical	Δ*H*_BDE_	Δ*H*_IP_	Δ*H*_PDE_	Δ*H*_PA_	Δ*H*_ETE_	Δ*H*_BDE_	Δ*H*_IP_	Δ*H*_PDE_	Δ*H*_PA_	Δ*H*_ETE_
^•^OCH_3_	−71	90	−161	−6	−65	−72	88	−160	−11	−61
^•^OC(CH_3_)_3_	−79	85	−164	−9	−70	−79	83	−163	−14	−66
^•^OH	−132	26	−158	−3	−129	−133	24	−157	−8	−125
^•^OOH	−5	81	−85	70	−74	−5	79	−84	65	−70
^•^OOCH_3_	11	125	−114	40	−29	10	124	−113	36	−25
^•^OO-CH = CH_2_	7	91	−84	71	−64	7	89	−83	66	−60
DPPH	30	37	−6	148	−118	30	35	−5	144	−114
O_2_^•^−	90	354	−264	126	−36	89	352	−263	122	−33

^a^Results represent mean values ± standard deviation (s.d.) of three independent measurements.

To determine the most probable mechanism of radical scavenging of **MB8** and **MB10** (hydrogen atom transfer (HAT), single electron—proton transfer (SET-PT) and sequential proton-loss electron-transfer (SPLET)), DFT was employed, homolytic/heterolytic dissociation of N–H bond was investigated. For estimation of the mechanism in the absence of free radicals, bond dissociation enthalpy (BDE), ionization potential (IP) and proton abstraction (PA) energies were calculated [13,54–60], while in the presence of free radicals, the reaction enthalpies (Δr*H*) were calculated for the reactions of **MB8** and **MB10** with each of the eight selected free radicals. Details on terms upon which radical selection was made (hydroxy (^•^OH), hydroperoxy (^•^OOH), methylperoxy (CH_3_–O–O^•^), superoxide radical anion (O_2_^•−^), methoxy (^•^OCH_3_), *tert*-butoxy (^•^OC(CH_3_)_3_), vinyl peroxy (CH_2_=CH–O–O^•^) and DPPH), as well as details on the calculation of thermodynamic parameters in the absence and in the presence of free radicals are provided in our previous studies, and for the sake of clarity presented in the electronic supplementary material [13,61]. Nevertheless, thermodynamical parameters and reaction enthalpies indicate which of HAT, SET-PT and SPLET mechanism prevails.

At first glance, based on the obtained high positive values for IP and Δ*H*_IP_, SET-PT is not a preferred mechanism for the radical scavenging. In the absence of free radicals, for both compounds, significantly lower values for PA than for BDE undoubtedly show that preferred antiradical mechanism is SPLET. On the other hand, enthalpies obtained for the reactions with free radicals point out HAT as a dominant antioxidative mechanism. Namely, for both **MB8** and **MB10**, values for Δ*H*_BDE_ are considerably lower than those obtained for Δ*H*_PA_. Similarly, as in the case of phenolic antioxidants, reaction enthalpies for the investigated aromatic amino carbonyl compounds are strongly influenced by nature and reactivity of quenched radical. The values for Δ*H*_BDE_ increase as reactivity of radical species decrease. The most exothermic reaction is in the case of ^•^OH radical, which is the most reactive among tested radicals. Somewhat higher, but still low Δ*H*_BDE_ values are obtained in the case of ^•^OCH_3_ and ^•^OC(CH_3_)_3_ radicals. Low negative or low positive reaction enthalpies for peroxy radicals indicate much slower reactions, while considerably endothermic nature of the reaction with O_2_^•−^ clearly shows that this radical will be poorly scavenged.

In addition to reaction enthalpies, change in Gibbs free energies was also inspected (electronic supplementary material, table S6). Obtained free energies indicate that there is almost no difference in reactivity obtained based on reaction enthalpies. The only deviation is in the case of PA of both **MB-8** and **MB-10**. Namely, as in the case of PA obtained from enthalpies (table 4), change in Gibbs free energy shows that SPLET will be preferred mechanism in the absence of free radicals and points out **MB-10** as a slightly better radical scavenger. On the other hand, results obtained based on Gibbs free energies indicate **MB-8** as a better radical scavenger.

Bearing in mind that for iodine atom relativistic effects are not negligible and using 6–311G(d,p) basis set might provide some inaccuracy, additional calculations with the larger def2-TZVP basis set were done (electronic supplementary material, table S7). The obtained results were almost the same as those calculated with 6–311G(d,p) basis set. This is most probably a consequence of the iodine atom being distanced from the reaction centre.

### 3.3. Mechanistic study for the formation of **MBs** in the presence of [HDEA][ClAc]

**Figure 2.**
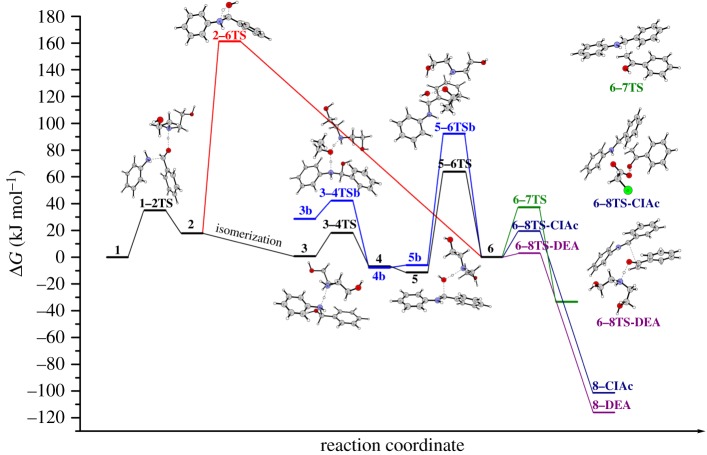
Energy diagrams of the pathways for the formation of **6** (the first phase) and for the C–C coupling (the second phase) of the studied mechanism.

**Scheme 2.**
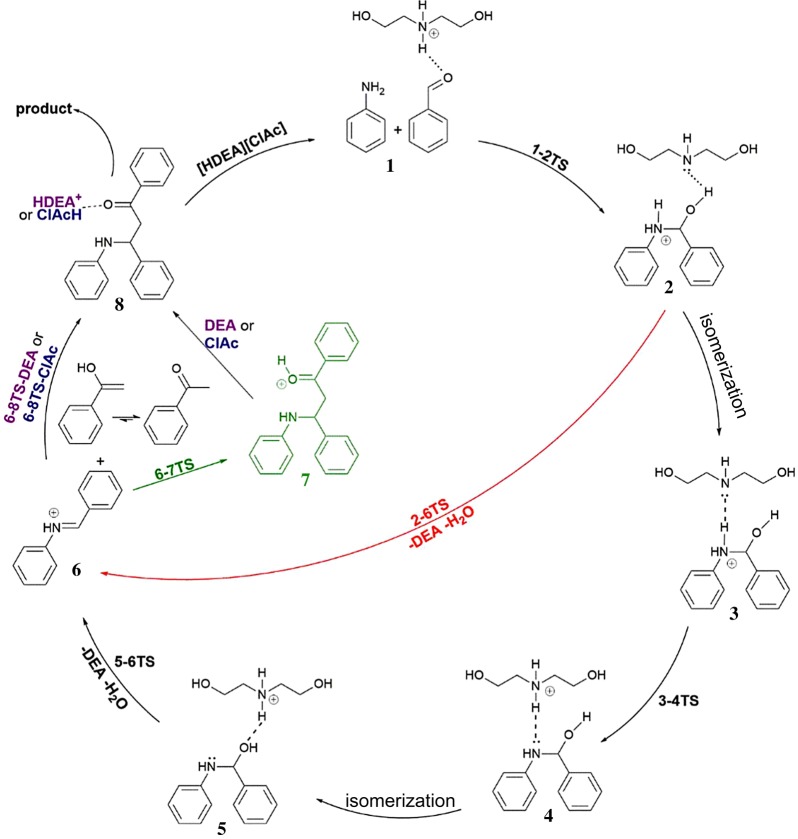
Proposed catalytic cycle of the examined Mannich reaction.

To explore the role of the catalyst in the reaction, the mechanistic study was performed using DFT. In our previous studies, we explored diastereoselectivity of the Mannich reaction catalysed with some ethanolamine-based ILs [62,63]. In those investigations, it was assumed that the catalyst takes active role only in dehydration step, while the C–C coupling is not catalysed. In this paper, mechanistic pathways of the organocatalysed reaction of acetophenone, benzaldehyde and aniline in the presence of [HDEA][ClAc] (figure 2, scheme 2) and pathways for the non-catalysed reaction (electronic supplementary material, figure S1) were investigated. A comparison of the structures and corresponding energies along these paths enables one to inspect the role of the catalyst in the first (C–N bond formation) and the second (C–C bond formation) steps. In the first step, the formation of iminium ion **6** via protonation of the substrates was inspected. Additionally, the influence of ethanol as a solvent molecule on the formation of iminium ion was explored. As far for the second phase of the reaction, i.e. C–C bond formation, we followed up the nucleophilic attack of the enol form of acetophenone to iminium ion **6**, in the presence of DEA and [ClAc], which are yielded during the reaction from [HDEA] [ClAc].

Reaction is initiated by the cationic part of the catalyst [HDEA], which contributes to the C–N bond formation via hydrogen bonding with the oxygen atom of the carbonyl group of benzaldehyde. Simultaneous nucleophilic attack of aniline nitrogen atom to the carbonyl group, along with the protonation of oxygen of carbonyl group of benzaldehyde by the catalyst (**1–2TS**) enables the formation of new C–N bond. We considered the possibility that C–N bond formation can be achieved with the support of [HDEA]. Namely, we assumed that [HDEA], as cationic part of the catalyst, and oxygen from carbonyl group of benzaldehyde can form a hydrogen bond, and as a consequence of N–H … O = C hydrogen bonding, the electrophilicity of carbonyl carbon atom will be increased. This assumption was confirmed with the NBO analysis of **1**. Comparison of the NBO charges in **1** and **SB-1** revealed that there is a slight increase in positive charge on this atom in **1** (electronic supplementary material, figure S2). Because of this, interatomic distances between the interacting carbon and nitrogen atoms are shortened. This unlocked the door for the concerted transition state **1–2TS**, where nitrogen of aniline performs a nucleophilic attack to the carbon atom of the carbonyl group. New C–N bond is being formed, and in the same time, a proton is being transferred from nitrogen atom of [HDEA] to the carbonyl oxygen atom. Carbonyl *π* bond is being broken and the electron pair is used for the O–H bond formation. Compared to the non-catalysed C–N bond formation (electronic supplementary material, figure S1), [HDEA] support resulted in significant decrease in the energetic demands for the C–N bond formation, with a necessary barrier of 35.0 kJ mol^−1^. Transition state **1–2TS** leads towards the formation of intermediate **2**. Here, C–N bond is completely formed, a proton is transferred from nitrogen atom of [HDEA] to the oxygen of carbonyl group. Consequentially, DEA is formed, and hydrogen bonded to the newly formed OH group (N…H–O hydrogen bond). When compared to the non-catalysed reaction, C–N coupling is energetically much more demanding, with an activation barrier of 149.6 kJ mol^−1^ (electronic supplementary material, figure S2). Intermediate **2** has two potential pathways for further transformations leading towards iminium ion **6**. The first one is with the participation of DEA, while the second one takes place without this molecule. In the reaction route where DEA is still present, **2** isomerizes to 17.1 kJ mol^−1^ more stable intermediate **3**. DEA molecule is now hydrogen bonded to N–H group of the same molecule (N … H–N hydrogen bond), enabling proton transfer to the nitrogen atom of DEA in transition state **3–4TS**. [HDEA] is formed and hydrogen bonded (N–H … N) to the amino alcohol **4**. This step of the reaction requires an activation barrier of 17.5 kJ mol^−1^. Intermediate **4** isomerizes into 4.3 kJ mol^−1^ more stable intermediate **5**, where [HDEA] is hydrogen bonded to the OH group of amino alcohol. This intermediate is suitable for the next step of the reaction, i.e. dehydration of amino alcohol. This transformation is presented by transition state **5–6TS** and requires 75.0 kJ mol^−1^. In this transition state, a proton is transferred from the nitrogen atom of [HDEA] to the oxygen atom of amino alcohol, while *σ* C–O bond is being broken. Formation of iminium ion **6** is accompanied by the liberation of water and DEA molecules. In the second possible transformation path, where DEA is liberated from the intermediate **2**, dehydration occurs via **2–6TS** without any assistance. Here, a proton is being transferred from aniline nitrogen to the benzaldehyde originating oxygen, *σ* C–O bond is being broken and water molecule liberated. This unassisted dehydration is energetically much more demanding, with an activation barrier of 143.6 kJ mol^−1^. Dehydration step in the non-catalysed reaction for the formation of the **SB** requires 222.9 kJ mol^−1^. Compared to the dehydration step in non-catalysed first step (figure 2), energetic demands are much lower in both catalysed pathways. However, direct transformation of **2** to iminium ion **6** is energetically discriminated by DEA catalysed process. In addition, iminium ion formation as an intermediate is in accordance with experimental findings of Suginome *et al*. that this ion serves as intermediate in Mannich-type reactions [64].

To test the influence of solvent along the reaction course for the formation of iminium ion **6**, a discrete molecule of ethanol was included (figure 2, blue line). This way, the influence of solvent on proton transfer and dehydration process was examined. Based on the obtained energies for these processes, one can see that ethanol did not significantly influence the deprotonation of the nitrogen atom in **3–4TSb**. On the other hand, isomerization of intermediate **4b** to **5b** is somewhat unfavoured, as well as dehydration process in **5–6TSb**. Namely, activation barrier is increased to 98.2 kJ mol^−1^. Additionally, the influence of solvent was explored on the formation of **SB**, in non-catalysed reaction (electronic supplementary material, figure S1). Here, the participation of solvent molecule is significantly lowering barriers for the formation of **SB**, making this reaction more competitive to the formation of iminium ion **6**.

Influence of DEA formed during the dehydration process in **5–6TS**, and [ClAc] anion on the C–C bond formation was also explored (figure 2 and scheme 2). First, we will examine C–C coupling without any assistance. It is worth pointing out that we examined similar C–C coupling in our previous studies, where we used cyclohexanone instead of acetophenone [62,63]. To keep the comparability of the non-catalysed and catalysed routes, we will briefly present the non-catalysed process. The first step of this phase of the reaction is a nucleophilic attack of enol form of acetophenone (methylene carbon) to the carbon atom of C = N bond of iminium ion **6**. This assumption was confirmed by revealing transition state **6–7TS**, with the necessary activation barrier of 37.2 kJ mol^−1^, respectively. In both cases, C = NH^+^ and enol C=C *π* bonds are being broken, electron pair from C=C bond is being used for the formation of new C–C bond, while the *π* electron pair from C = NH^+^ is being placed on the nitrogen atom. This way, intermediate **7**, a protonated final product of the reaction, is formed. Unfortunately, we were unable to locate corresponding transition states for the deprotonation of these intermediates, either by DEA or ClAc. Nevertheless, we located intermediates **8-DEA** and **8-ClAc**, which would be yielded from such process.

To examine the influence of DEA and [ClAc] in this phase of the reaction, we included these species in the interaction which would produce a new C–C bond. To be precise, we assumed that bases DEA, with ‘free’ electron pair situated on the nitrogen atom, and [ClAc] anion, can establish hydrogen bonds with hydrogen from OH group of enol in **6**. This was confirmed by revealing the structures of **6 DEA** and **6** [**ClAc**]. Next reaction step takes place with the simultaneous formation of C–C bond, and proton transfer from enol oxygen to the hydrogen bond acceptor atom of each base. In the same time, *π* C=N and C=C (enol) bonds are being broken, electron pair from *π* C=N bond is being placed on the nitrogen atom of C=N, while *π* C=C electron pair of enol is used for new C–C bond formation with **6**. O–H bond is being broken, and proton transferred to the base. Necessary barriers for **6–8TS-DEA** and **6–8TS-ClAc** amount to 2.9 and 19.4 kJ mol^−1^. For the difference with the non-catalysed process of C–C bond formation, there is no formation of protonated final product **7**. Instead, in **8-DEA** and **8-ClAc** proton is completely transferred to the corresponding acceptor atoms of each base (nitrogen of DEA, or oxygen of [ClAc]). This way, the final product of the reaction **MB1** is liberated, and cationic part of the catalyst [HDEA] or chloroacetic acid is formed. In such manner, in the case of DEA, the catalyst is regenerated, while in case of [ClAc] precursor of the catalyst is formed.

One can note that the presence of [ClAc] slightly lowered C–C coupling activation barriers, while DEA significantly decreased these activation energies. Obtained results clearly point out that the rate-determining step of the overall reaction is water molecule elimination in **5–6TS** transition state. It is worth pointing out that, especially in the case of the reactions with anilines substituted with methyl and methoxy group, beside the formation of Mannich product, formation of the **SB** was detected. It might be rationalized in the way that in **SB** formation process solvent is taking some part in the reaction by lowering the barrier for proton transfer and therefore making this reaction more competitive to the first phase of Mannich reaction, i.e. formation of the iminium ion.

## 4. Conclusion

Three-component Mannich reaction of benzaldehyde, anilines and acetophenone, or 4-iodoacetophenone, was performed in the presence of [HDEA][ClAc]. All products were obtained in moderate to high yields. Five of them (**MB1–MB3**, **MB9** and **MB10**) are newly synthetized compounds, firstly reported in this paper. In all experimental UV–Vis spectra of **MB1**–**MB10**, there are two major absorption bands around 205 and 260 nm, as well as a small band around 310 nm. Unlike numerous studies, here B3LYP method failed in the reproduction of the experimental spectra. On the other hand, M052X method reproduced all experimental spectra.

*In vitro* DPPH radical assay showed that among all investigated compounds, only compounds with anisidine moiety (**MB8** and **MB10**) express moderate activity. Results obtained for thermodynamic parameters point out SPLET as preferred radical quenching mechanism. On the other hand, enthalpies for the reactions are strongly dependent on the nature of radical species. Nevertheless, considerably lower values for Δ*H*_BDE_ than those obtained for Δ*H*_PA_ point out HAT as preferred mechanism to SPLET in the presence of free radicals, and in methanol as solvent.

Based on the results obtained from the mechanistic study, one can conclude that hydrogen-bonded organocatalysis plays a very important role in both phases of the reaction. Rate-limiting step of the entire reaction is water molecule elimination in **5–6TS** transition state. The importance of the hydrogen bonding in this reaction is reflected in the findings that the presence of [HDEA] significantly lowered the barrier for the formation of the new C–N bond. DEA, formed during this process, enhances proton transfer from nitrogen to oxygen. That way, barriers for the formation of iminium ion are significantly lowered. In addition, the final phase of the reaction, i.e. simultaneous formation of the new C–C bond and deprotonation of the oxygen atom, is also promoted by either DEA or [ClAc]. On the other hand, the effect of ethanol, as solvent and co-catalyst in proton transfer, is more pronounced in the formation of **SB**, than in processes for the formation of iminium ion **6**.

## Supplementary Material

Supporting data for the main document

## Data accessibility

The datasets supporting this article have been uploaded as part of the electronic supplementary material.
