# Supplementary material for: Acetophenone Mannich bases: study of ionic liquid catalysed synthesis and antioxidative potential of products
Source: R Soc Open Sci. 2018 Nov 14;5(11):181232. doi: 10.1098/rsos.181232 (PMC6281942; doi:10.1098/rsos.181232)
Supplement: Supporting data for the main document [file rsos181232supp1.docx]

**Electronic Supplementary Material**

**Acetophenone Mannich bases: Study of ionic liquid catalysed synthesis and antioxidative potential of products**

***Vladimir P. Petrović,^†a^ Dušica Simijonović,^a^ Vesna M. Milovanović^a^ and Zorica D. Petrović^a^***

**^a^University of Kragujevac, Faculty of Science, Department of Chemistry, Kragujevac, Serbia**

Corresponding author’s e-mail address: [vladachem@kg.ac.rs](mailto:vladachem@kg.ac.rs)

University of Kragujevac, Faculty of Science, 12 Radoja Domanovića, 34000 Kragujevac, Serbia, Tel.: ++(381) 34 335 039

**Table of contents**

[**Figure S1**. Energy diagram of the non-catalyzed (black lines) and ethanol assisted reactions (red lines) for the formation of Schiff base **SB.** 3](#_Toc525119959)

[**Figure S2**. The optimised structures of **SB**-**1**, **1**, and **2**, with delineated crucial NBO charges on specific atoms and interatomic distances (Å). 4](#_Toc525119960)

[**Figure S3**. Experimental and simulated UV-Vis spectra of **MB2 – MB10**. 5](#_Toc525119961)

[**Figure S4**. Kohn–Sham orbitals of **MB1**. Isovalues used for depiction of orbitals in all cases MO=0.02 and Density=0.0004. 7](#_Toc525119962)

[**Figure S5**. Kohn–Sham orbitals of **MB2**. 9](#_Toc525119963)

[**Figure S6**. Kohn–Sham orbitals of **MB3**. 11](#_Toc525119964)

[**Figure S7**. Kohn–Sham orbitals of **MB4**. 12](#_Toc525119965)

[**Figure S8**. Kohn–Sham orbitals of **MB5**. 14](#_Toc525119966)

[**Figure S9**. Kohn–Sham orbitals of **MB6**. 16](#_Toc525119967)

[**Figure S10**. Kohn–Sham orbitals of **MB7**. 18](#_Toc525119968)

[**Figure S11**. Kohn–Sham orbitals of **MB8**. 20](#_Toc525119969)

[**Figure S12**. Kohn–Sham orbitals of **MB9**. 21](#_Toc525119970)

[**Figure S13**. Kohn–Sham orbitals of **MB10**. 23](#_Toc525119971)

[**Table S1.** Electron transitions responsible for the appearance of bands in UV-Vis spectra of compounds **MB1**-**MB5.** 24](#_Toc525119972)

[**Table S2.** Electron transitions responsible for the appearance of bands in UV-Vis spectra of compounds **MB6**-**MB10.** 25](#_Toc525119973)

[**Table S3.** Energies (a.u.) of orbitals of compounds **MB1**-**MB5**. 26](#_Toc525119974)

[**Table S4.** Energies (a.u.) of orbitals of compounds **MB6**-**MB7**. 27](#_Toc525119975)

[**Table S5.** Interaction of the examined and reference compounds with the stable radical DPPH. 28](#_Toc525119976)

[**Table S6.** Calculated thermodynamical parameters (kJ mol^-1^) of antioxidant mechanisms for **MB-8** and **MB-10**, and reaction energies (kJ mol^-1^) for the reactions of these compounds with the selected radicals in methanol based on Gibbs free energies. 28](#_Toc525119977)

[**Table S7.** Calculated thermodynamical parameters (kJ mol^-1^) of antioxidant mechanisms for **MB-8** and **MB-10**, and reaction enthalpies (kJ mol^-1^) for the reactions of these compounds with the selected radicals in methanol. M052X method and 6-311++G(d,p) basis set were used for all atoms except of iodine, where def2-TZVP basis set was used, and the conductor like solvation model (CPCM). 29](#_Toc525119978)

[Details for calculations of thermodynamical parameters 30](#_Toc525119979)

[Thermodynamical parameters in the absence of free radical species 30](#_Toc525119980)

[Thermodynamic parameters in the presence of harmful free radicals 31](#_Toc525119981)

[Spectral characterization of compounds **MB4**-**MB8** 33](#_Toc525119982)

[^1^H and ^13^C NMR spectra of **MB1** 34](#_Toc525119983)

[^1^H and ^13^C NMR spectra of **MB2** 35](#_Toc525119984)

[^1^H and ^13^C NMR spectra of **MB3** 36](#_Toc525119985)

[^1^H and ^13^C NMR spectra of **MB4** 37](#_Toc525119986)

[^1^H and ^13^C NMR spectra of **MB5** 38](#_Toc525119987)

[^1^H and ^13^C NMR spectra of **MB8** 41](#_Toc525119988)

[^1^H and ^13^C NMR spectra of **MB9** 42](#_Toc525119989)


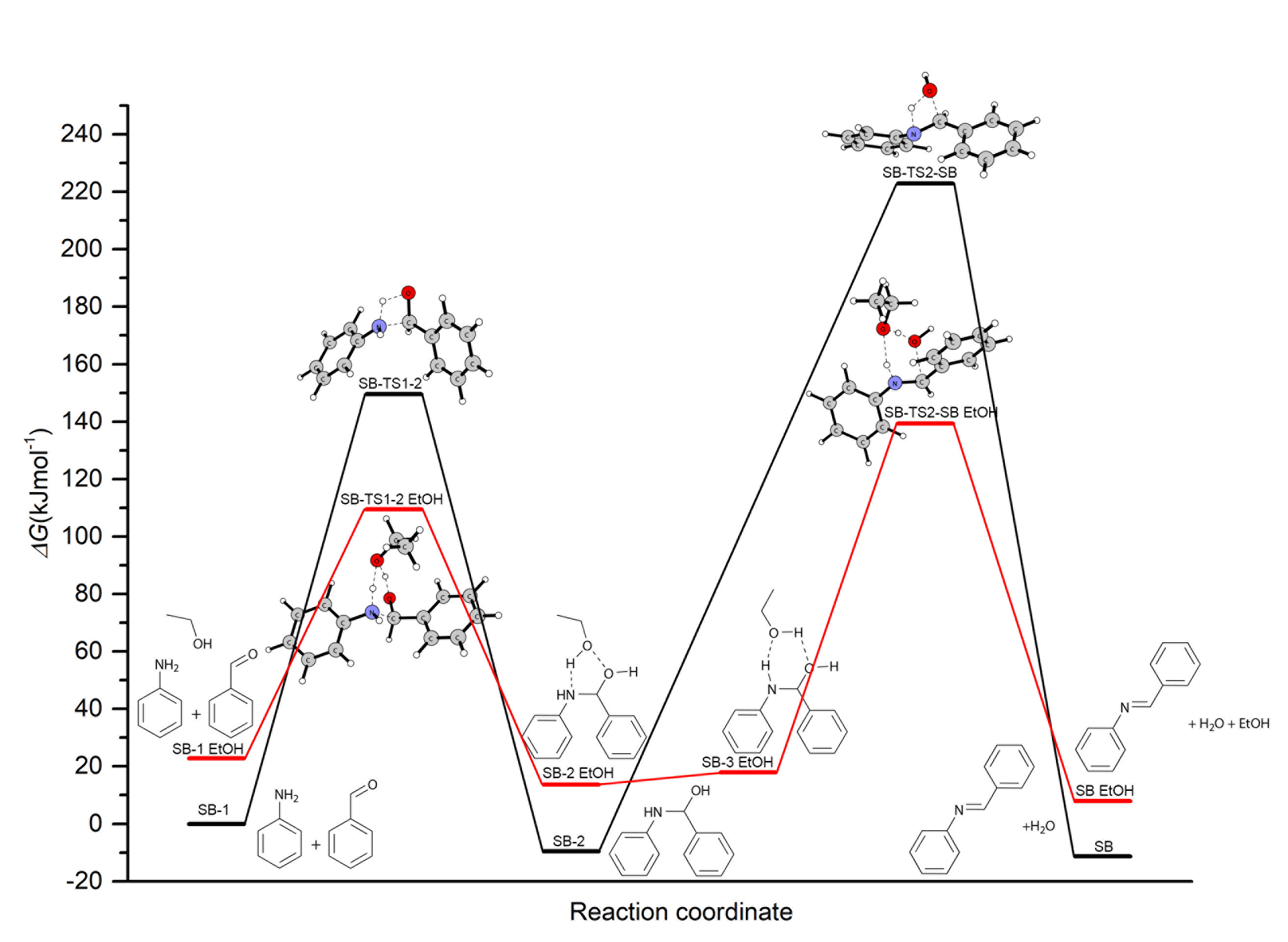


# **Figure S1**. Energy diagram of the non-catalyzed (black lines) and ethanol assisted reactions (red lines) for the formation of Schiff base **SB.**


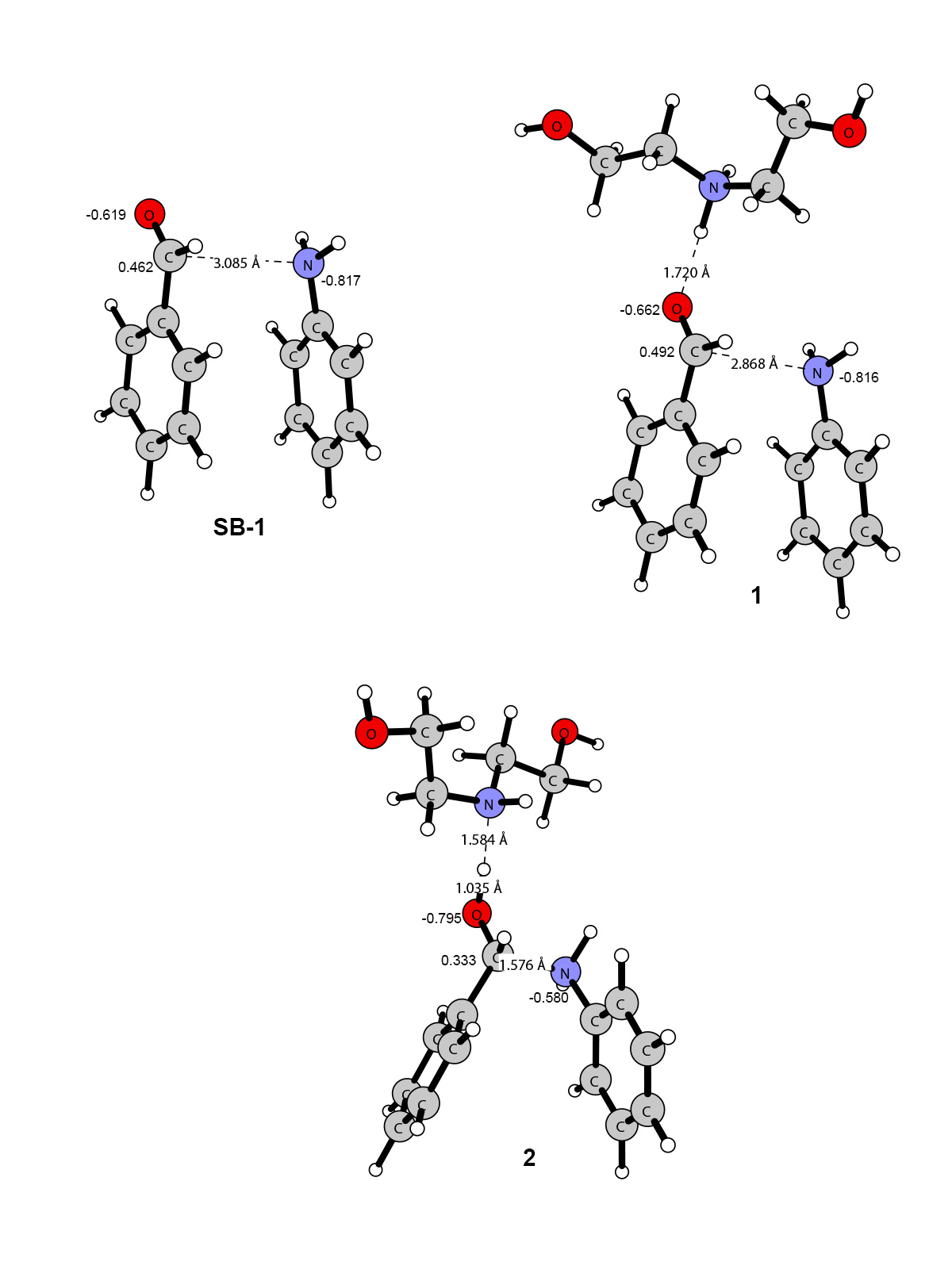


# **Figure S2**. The optimised structures of **SB**-**1**, **1**, and **2**, with delineated crucial NBO charges on specific atoms and interatomic distances (Å).


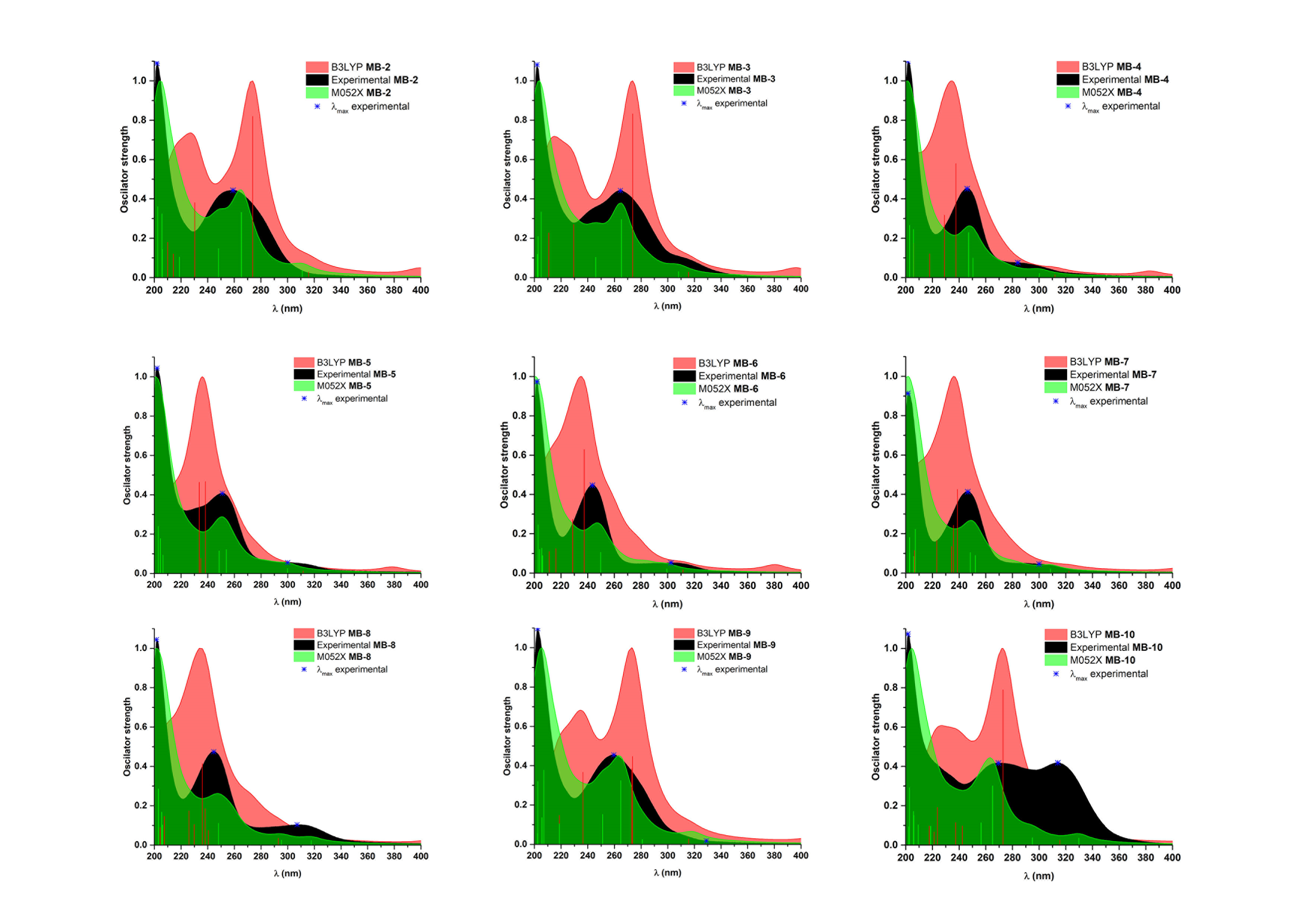


# **Figure S3**. Experimental and simulated UV-Vis spectra of **MB2 – MB10**.

| HOMO 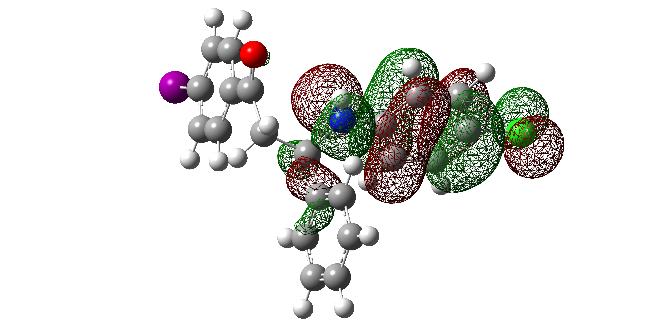 | LUMO 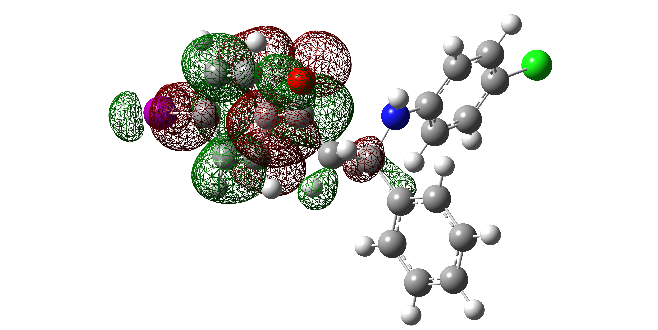 |
| --- | --- |
| HOMO-2 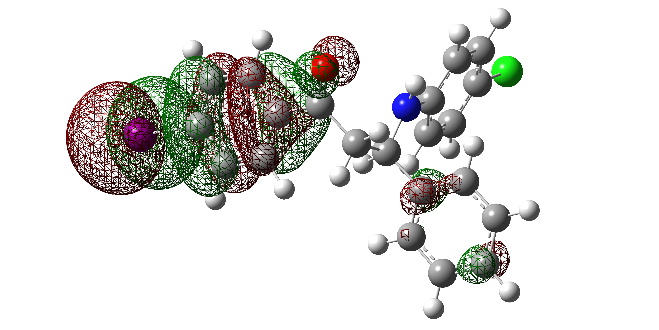 | LUMO+1 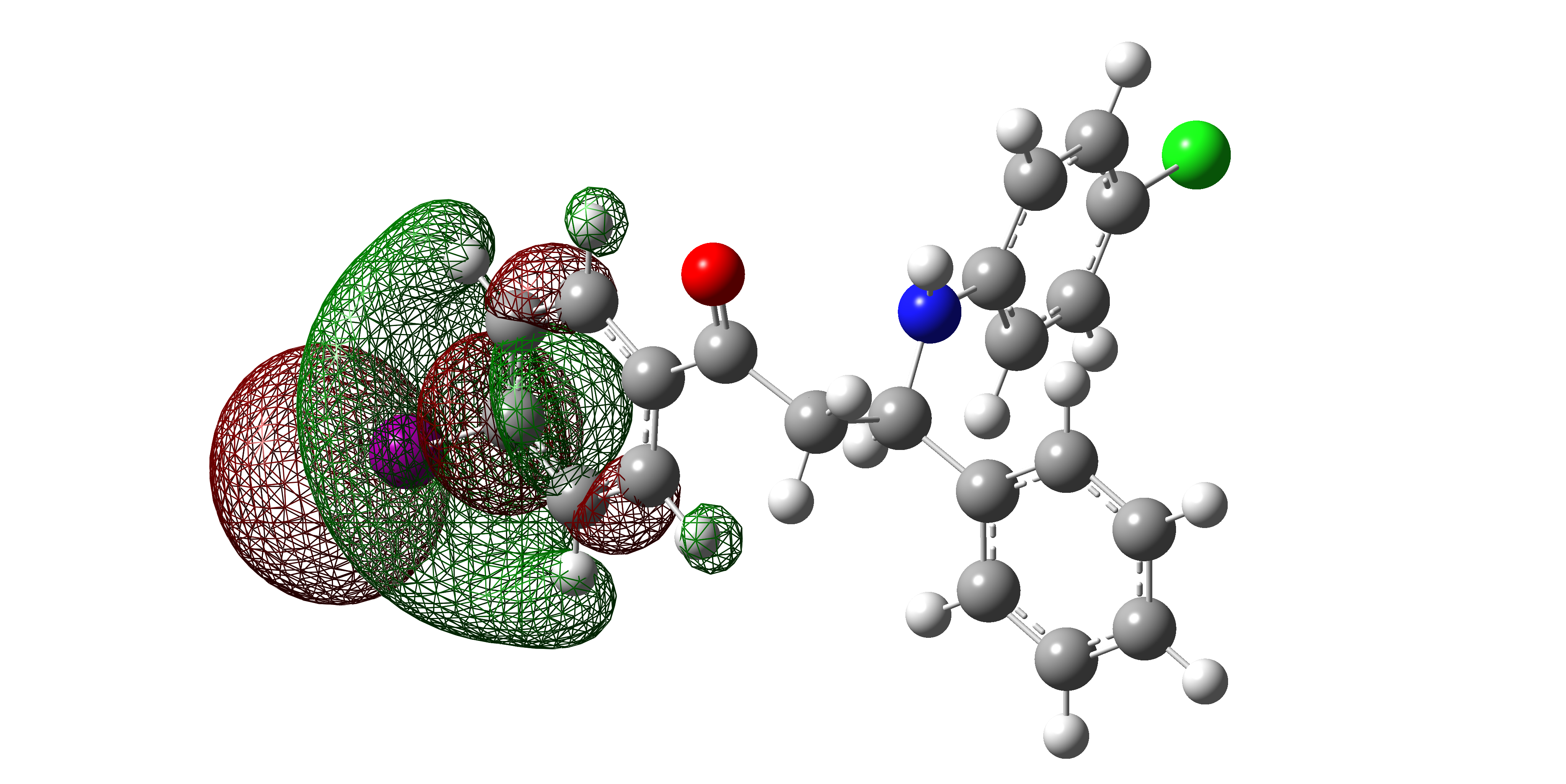 |
| HOMO-3 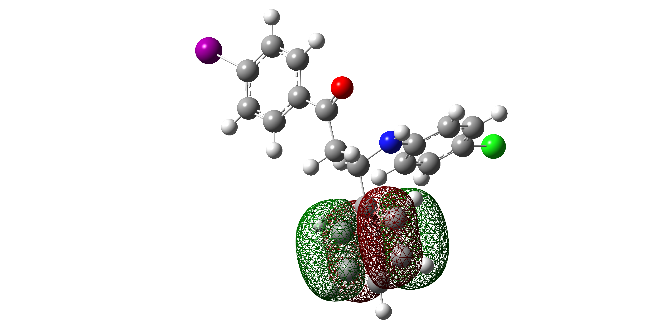 | LUMO+2 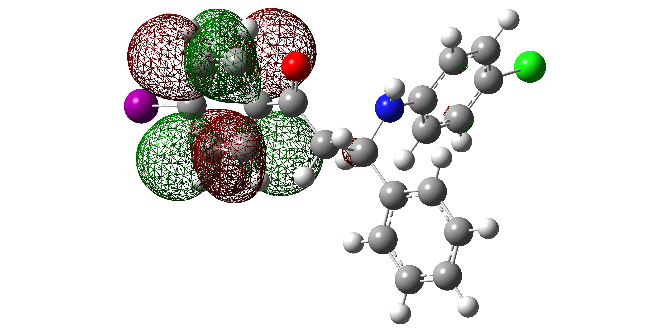 |
| HOMO-4 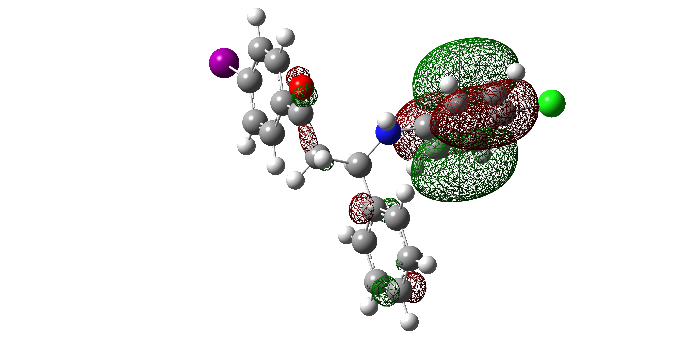 | LUMO+3 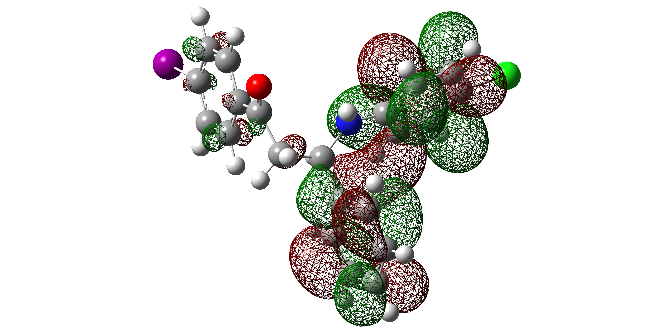 |
| HOMO-5 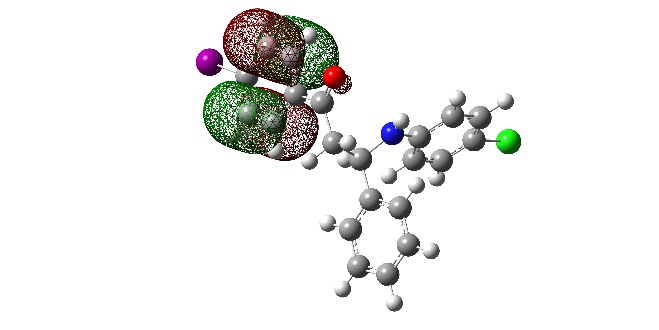 | LUMO+4 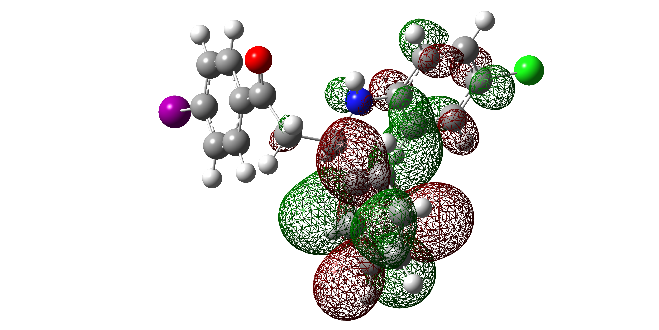 |
|  | LUMO+5 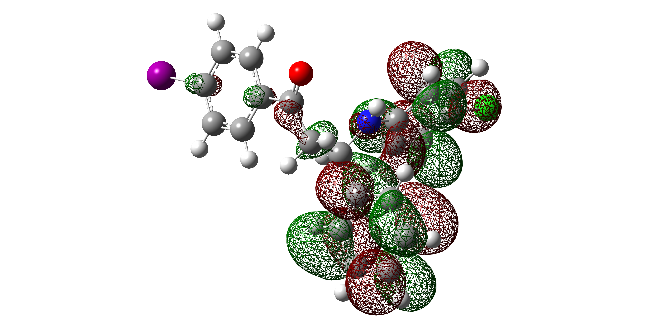 |

# **Figure S4**. Kohn–Sham orbitals of **MB1**. Isovalues used for depiction of orbitals in all cases MO=0.02 and Density=0.0004.

| HOMO 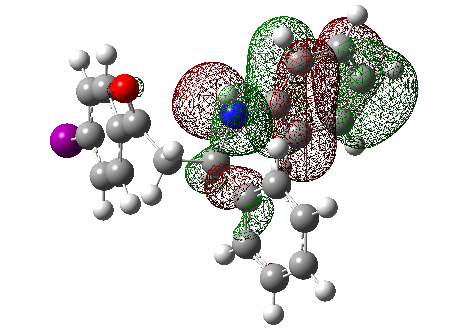 | LUMO 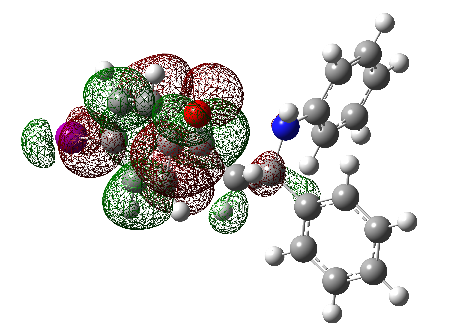 |
| --- | --- |
| HOMO-1 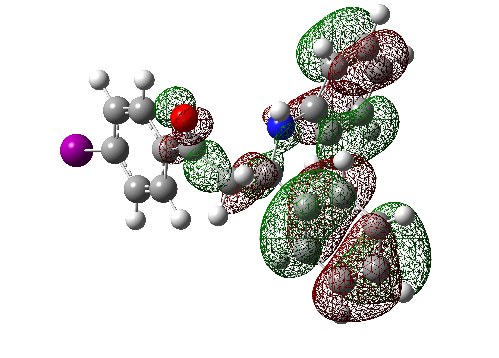 | LUMO+2 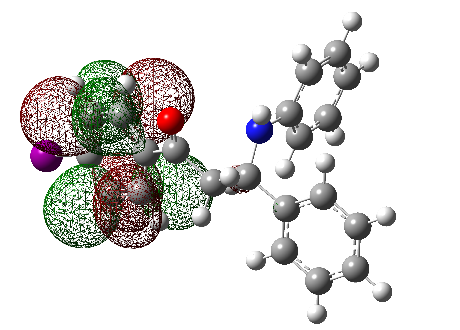 |
| HOMO-2 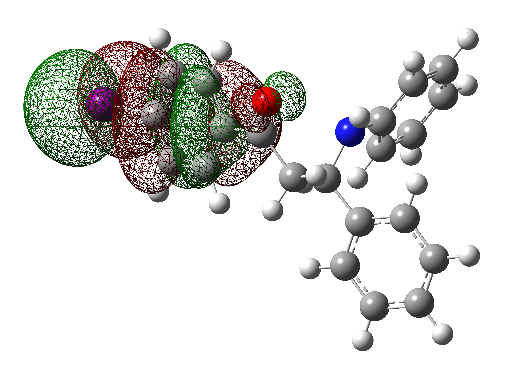 | LUMO+3 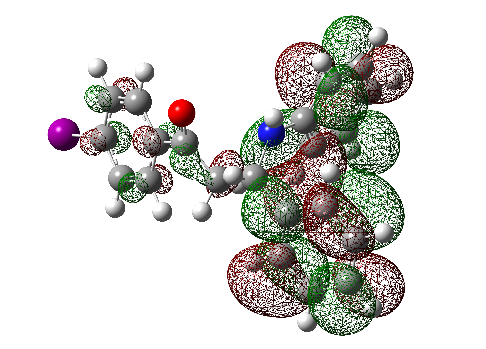 |
| HOMO-3 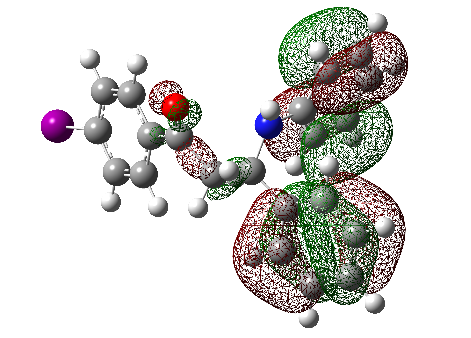 | LUMO+4 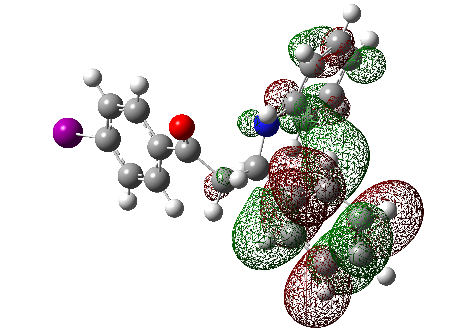 |
| HOMO-4 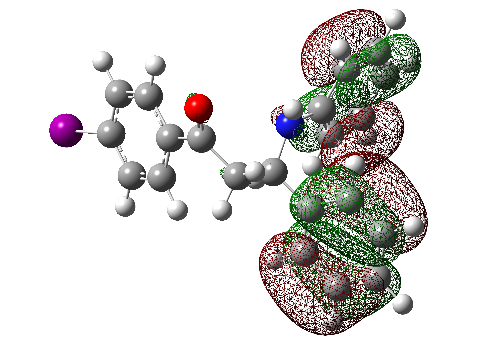 | LUMO+5 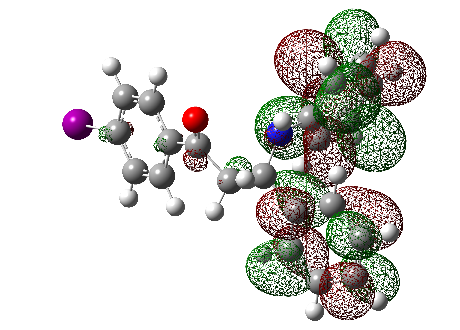 |
| HOMO-5 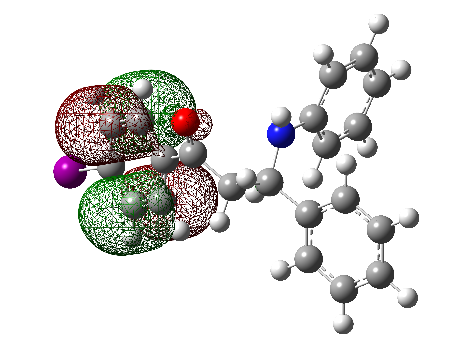 | LUMO+7 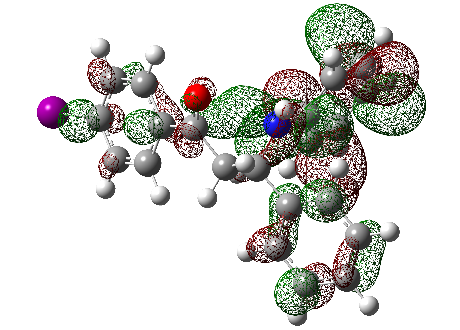 |
| HOMO-8 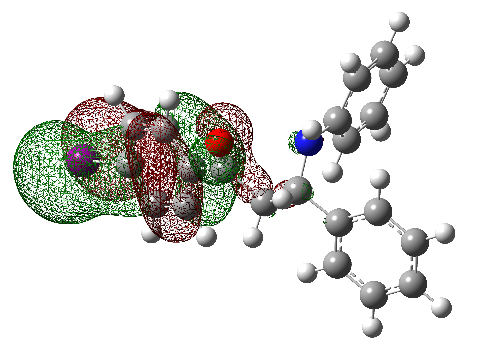 |  |

# **Figure S5**. Kohn–Sham orbitals of **MB2**.

| HOMO 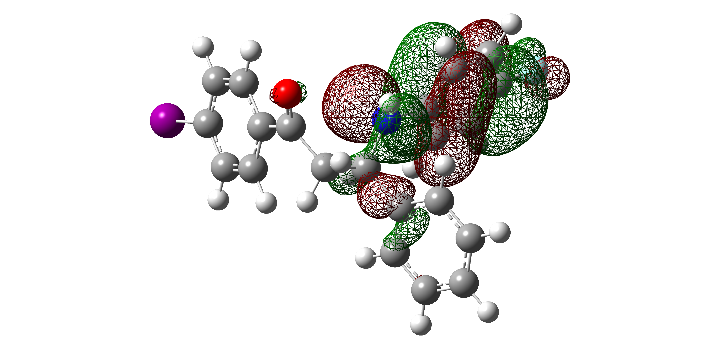 | LUMO 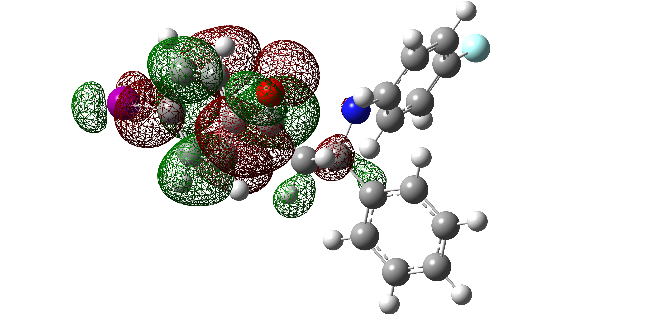 |
| --- | --- |
| HOMO-1 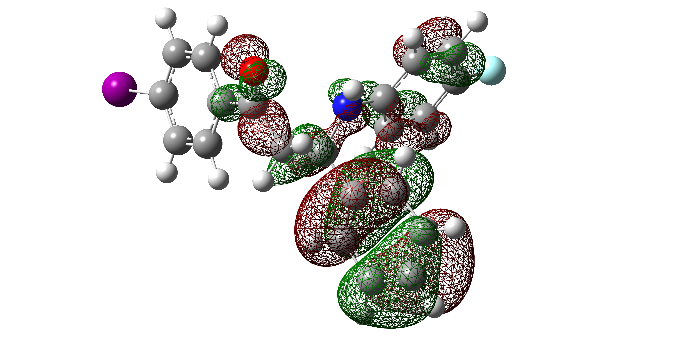 | LUMO+2 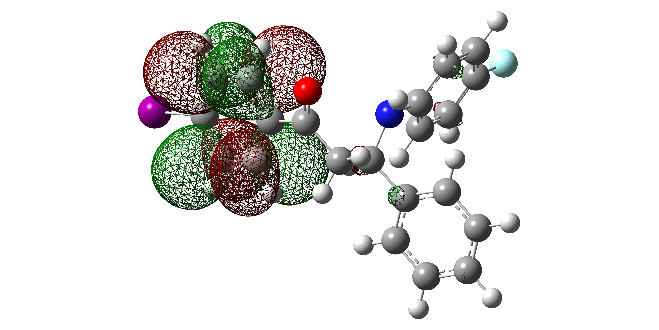 |
| HOMO-2 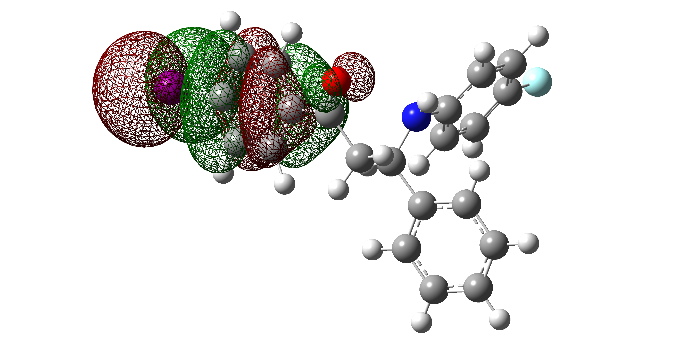 | LUMO+3 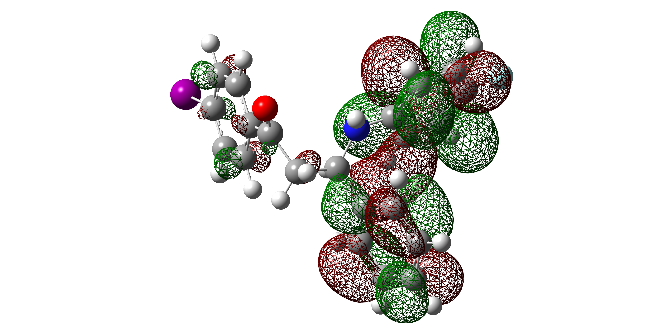 |
| HOMO-4 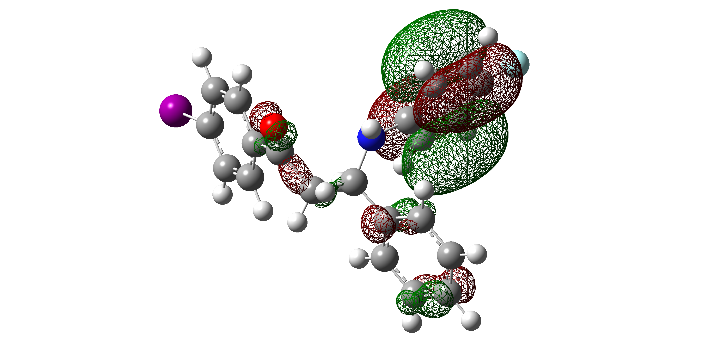 | LUMO+4 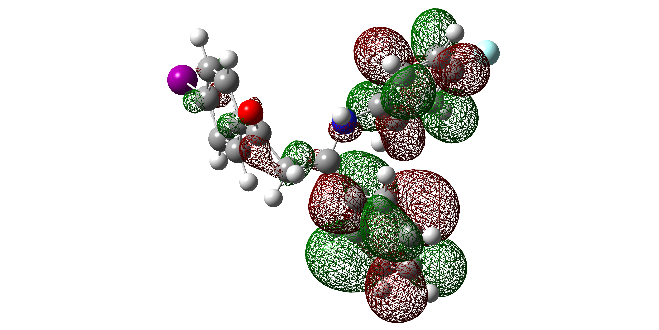 |
| HOMO-5 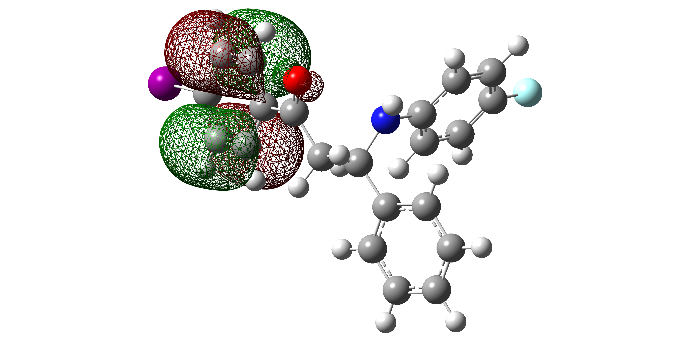 | LUMO+5 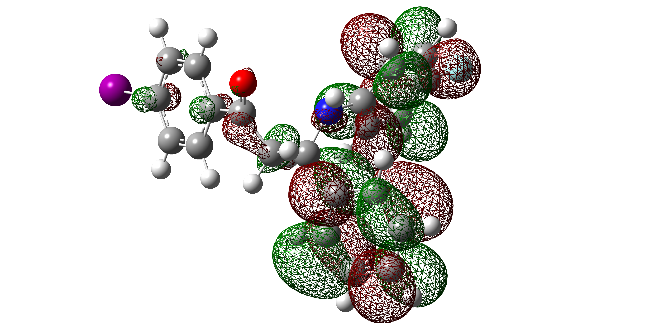 |
|  | LUMO+8 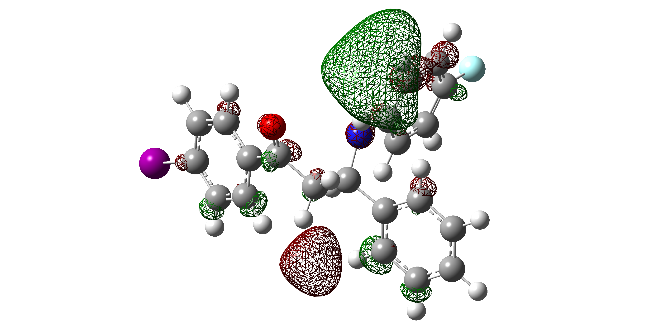 |

# **Figure S6**. Kohn–Sham orbitals of **MB3**.

| HOMO 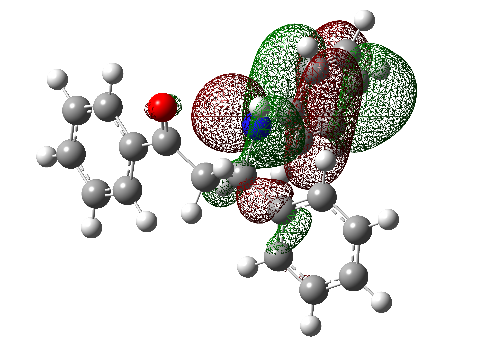 | LUMO 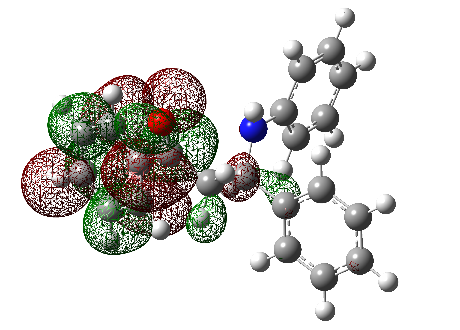 |
| --- | --- |
| HOMO-2 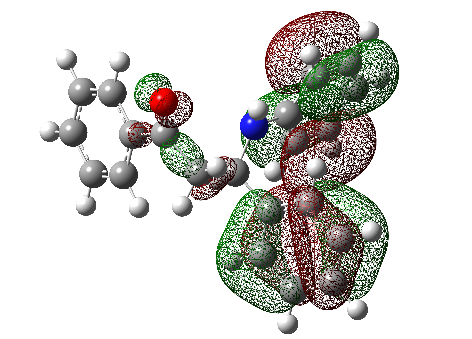 | LUMO+1 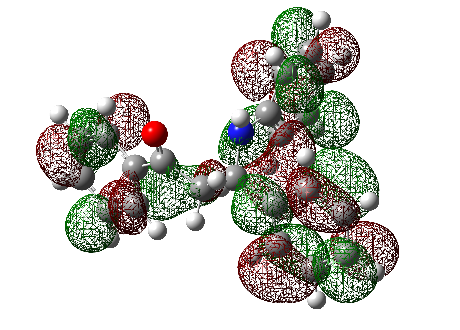 |
| HOMO-3 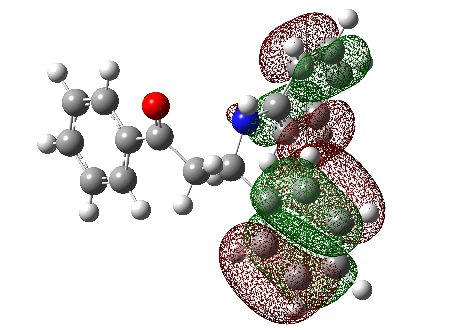 | LUMO+2 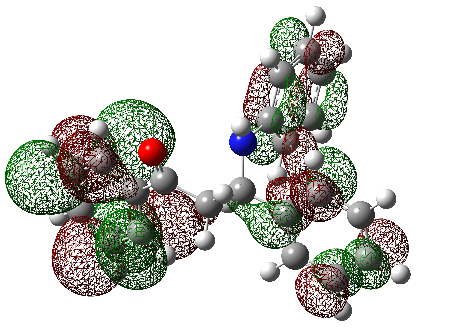 |
| HOMO-4 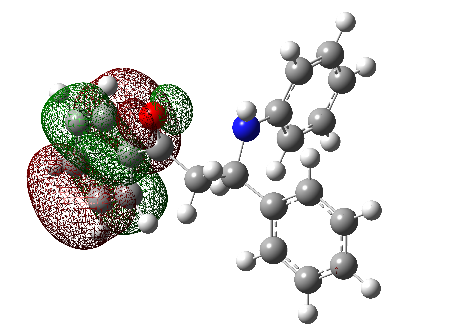 | LUMO+3 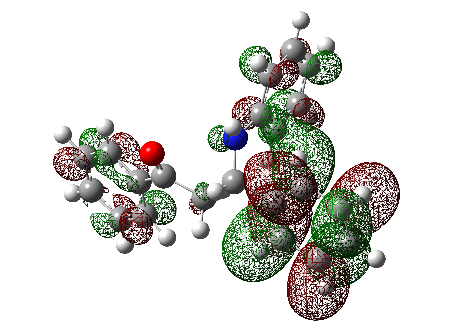 |
| HOMO-5 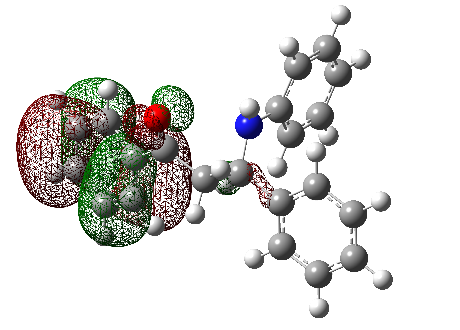 | LUMO+4 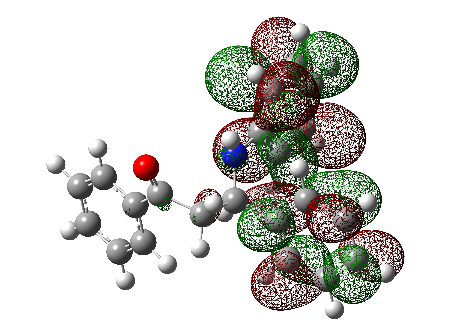 |

# **Figure S7**. Kohn–Sham orbitals of **MB4**.

| HOMO 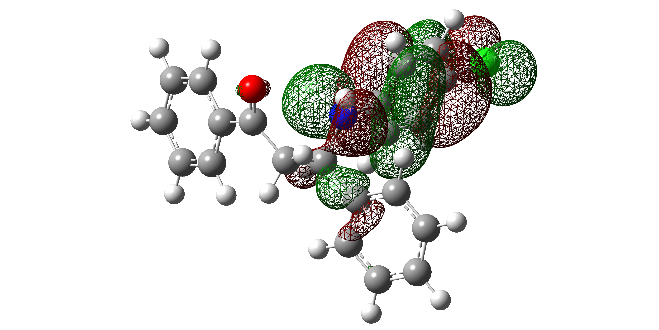 | LUMO 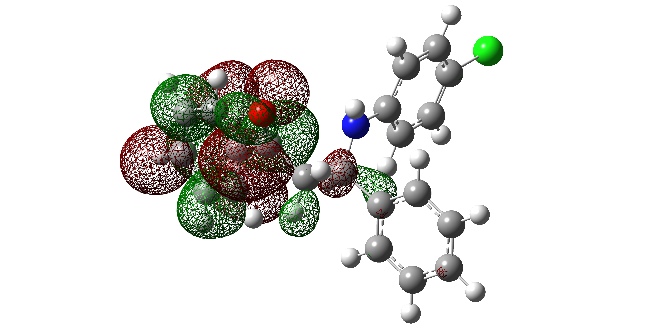 |
| --- | --- |
| HOMO-1 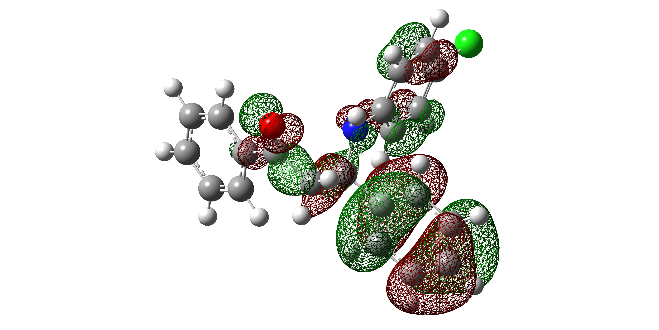 | LUMO+1 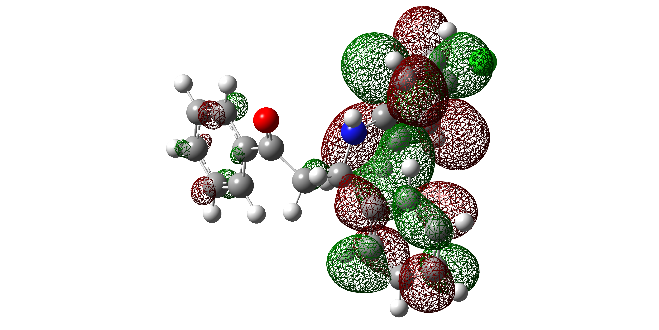 |
| HOMO-3 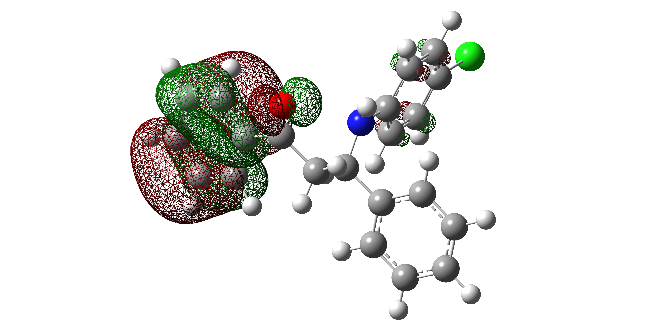 | LUMO+2 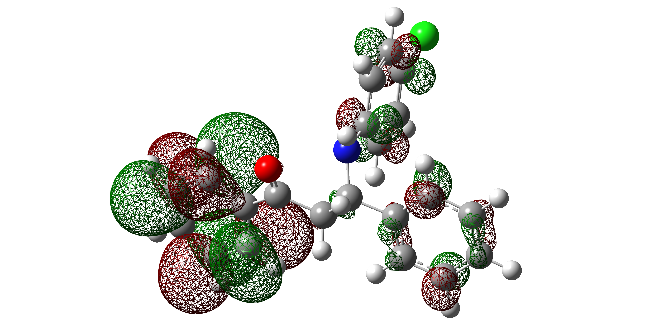 |
| HOMO-4 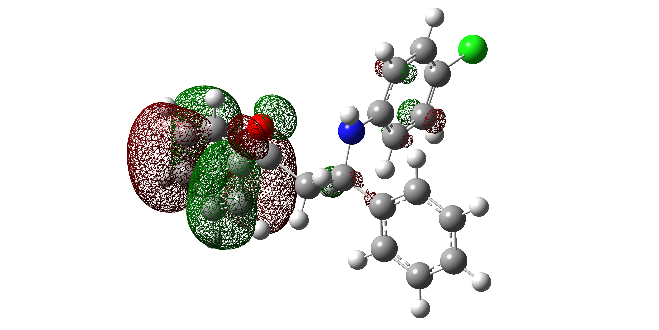 | LUMO+3 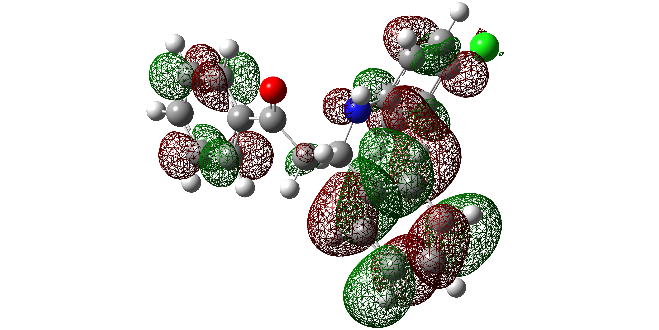 |
| HOMO-5 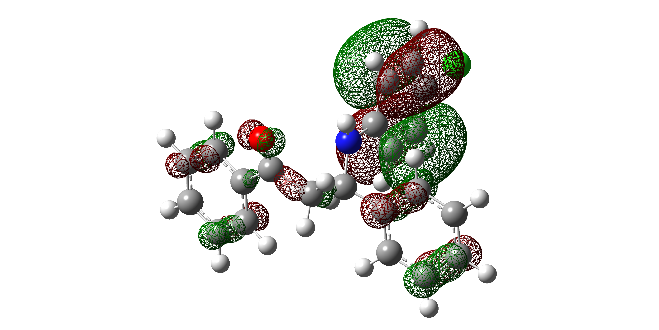 | LUMO+5 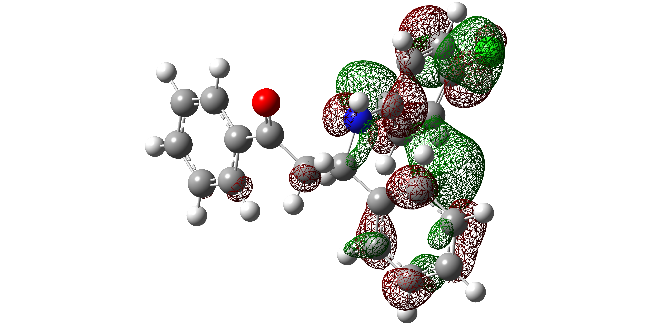 |
|  | LUMO+7 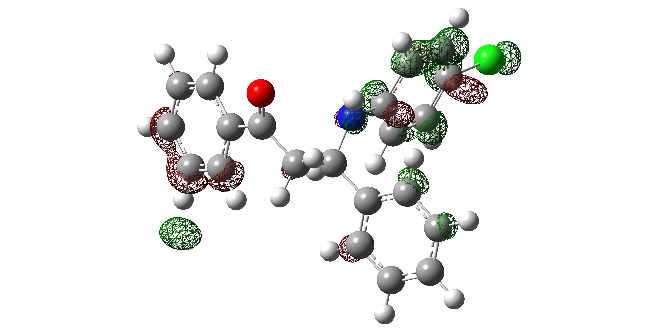 |

# **Figure S8**. Kohn–Sham orbitals of **MB5**.

| HOMO 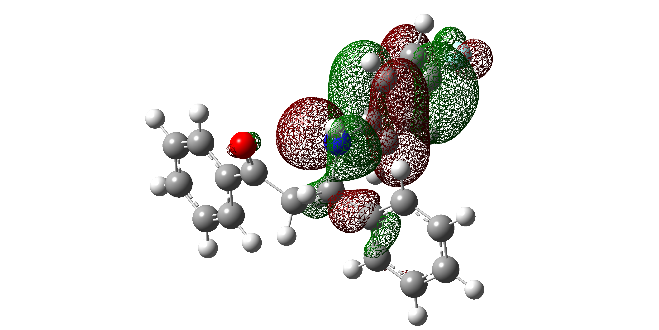 | LUMO 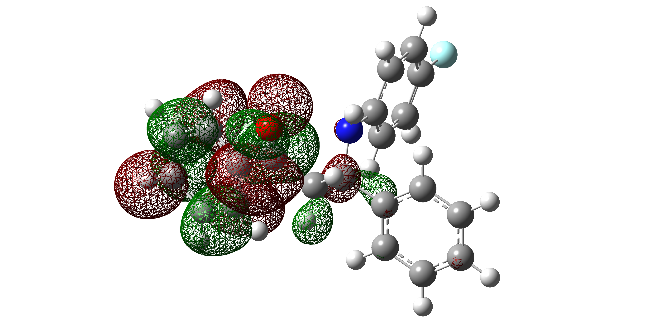 |
| --- | --- |
| HOMO-1 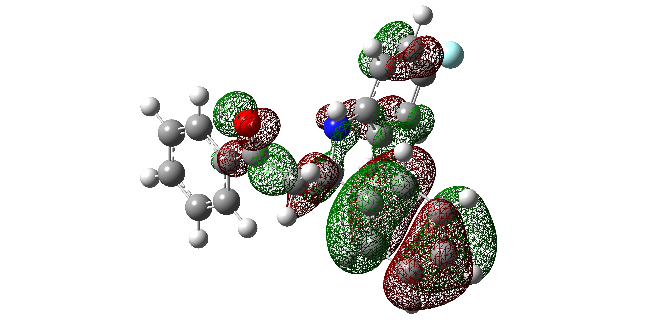 | LUMO+1 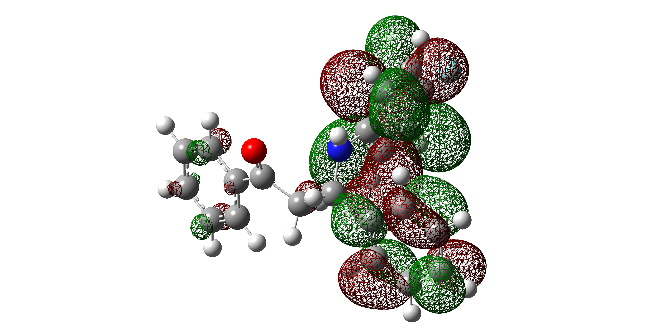 |
| HOMO-2 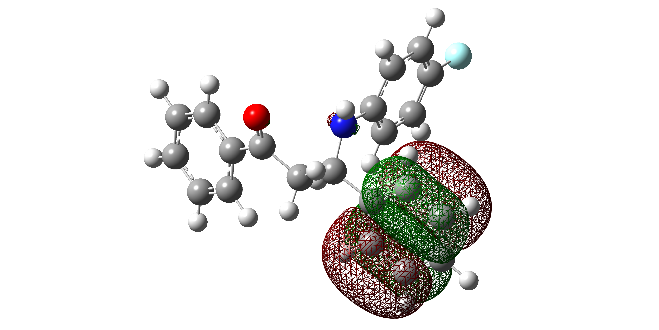 | LUMO+2 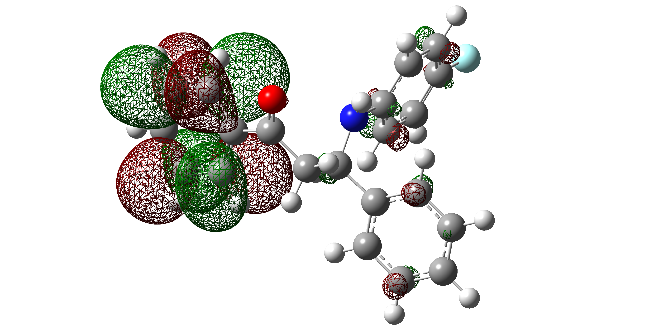 |
| HOMO-3 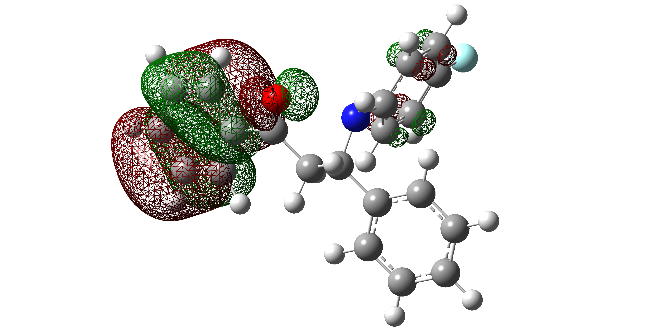 | LUMO+3 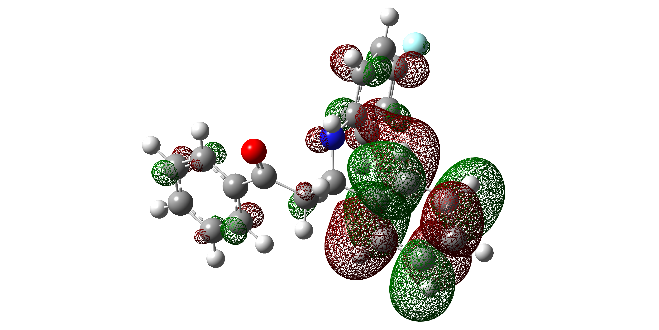 |
| HOMO-4 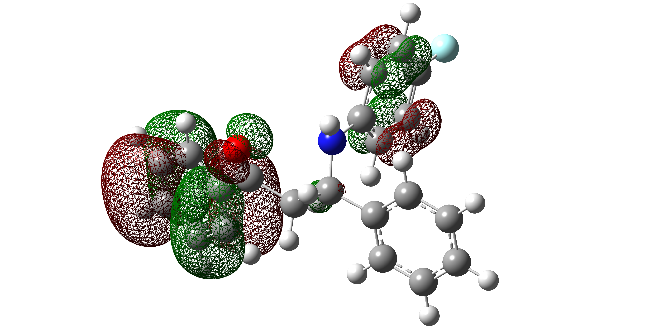 |  |
| HOMO-5 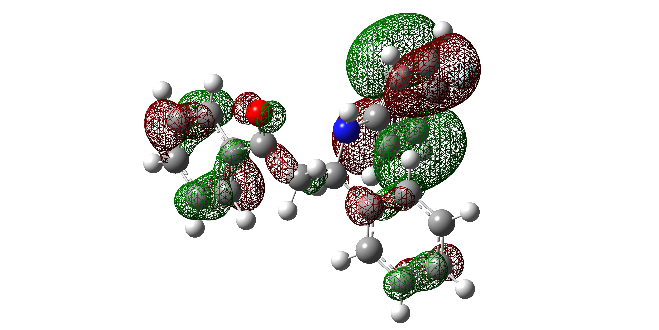 |  |

# **Figure S9**. Kohn–Sham orbitals of **MB6**.

| HOMO 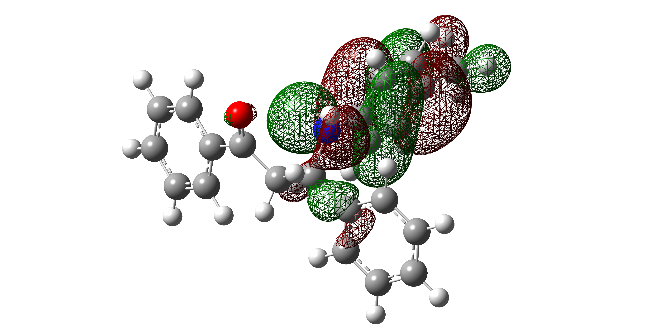 | LUMO 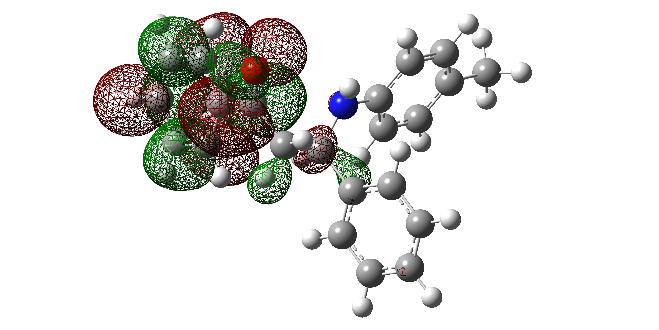 |
| --- | --- |
| HOMO-1 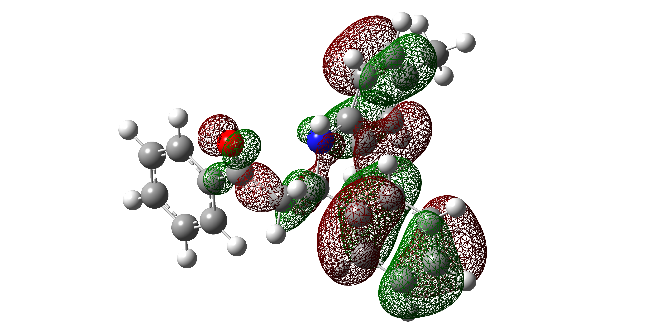 | LUMO+1 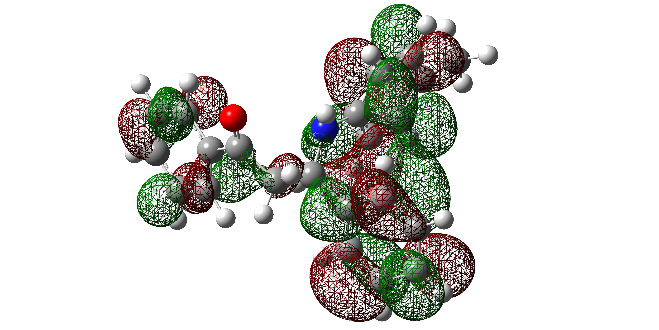 |
| HOMO-2 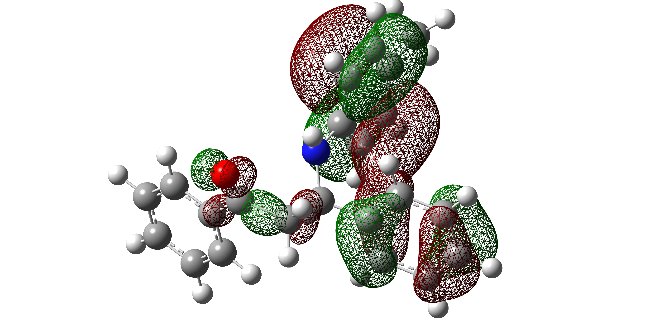 | LUMO+2 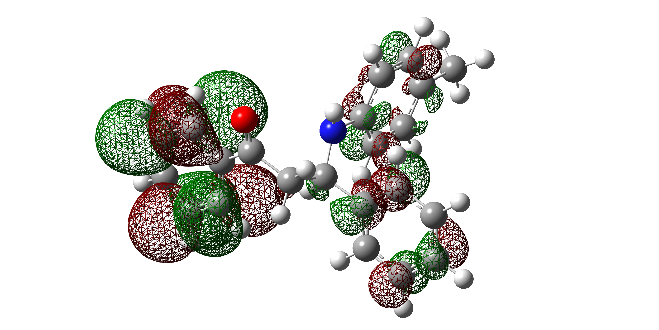 |
| HOMO-4 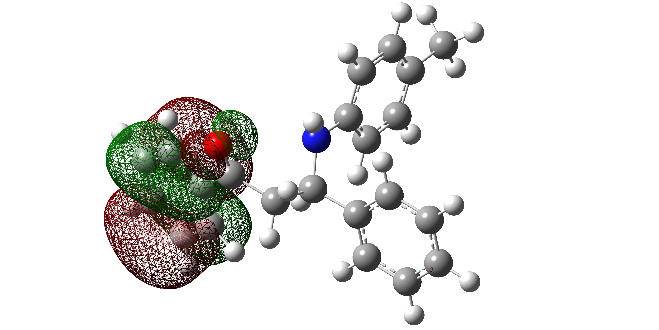 | LUMO+3 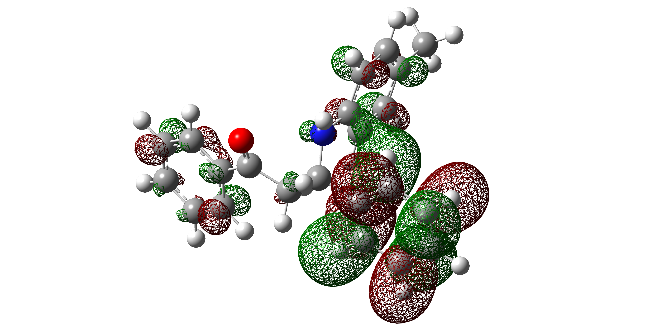 |
| HOMO-5 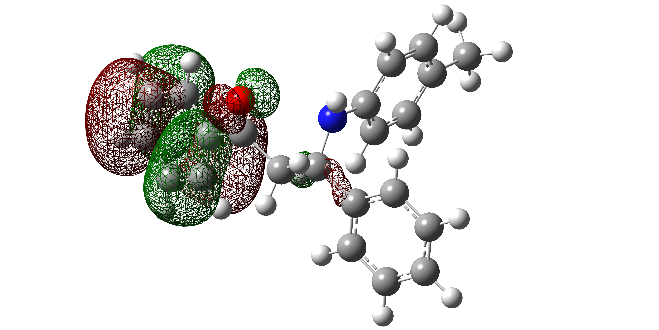 | LUMO+4 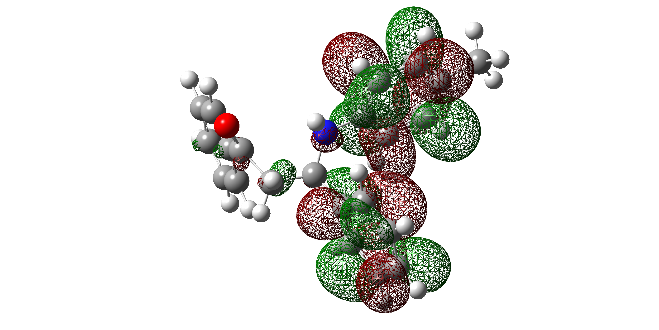 |
| HOMO-6 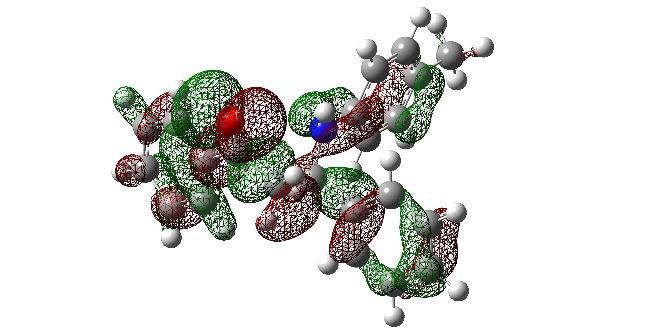 | LUMO+6 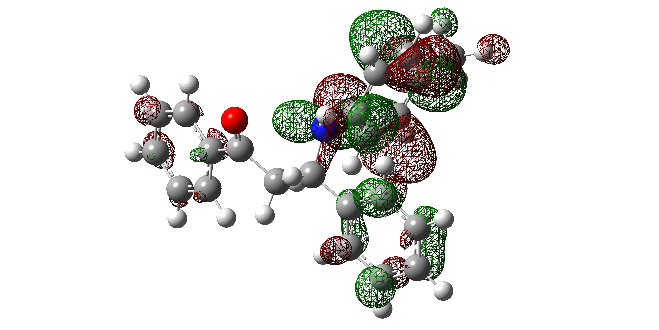 |
|  | LUMO+7 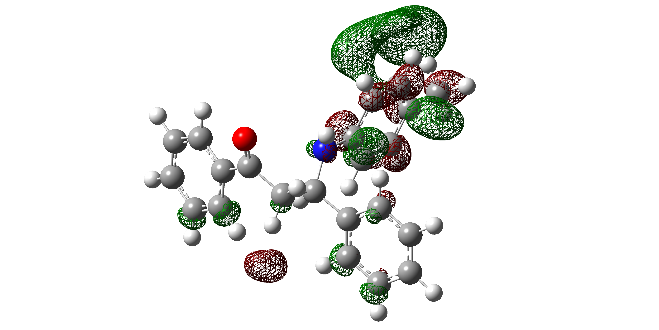 |
|  | LUMO+8 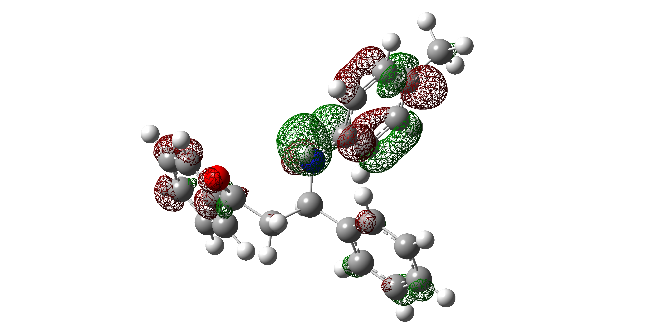 |
|  | LUMO+9 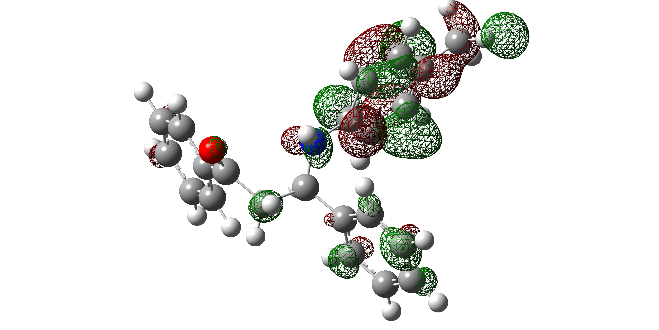 |

# **Figure S10**. Kohn–Sham orbitals of **MB7**.

| HOMO 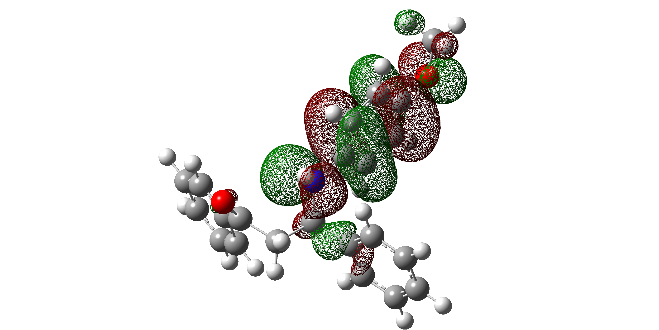 | LUMO 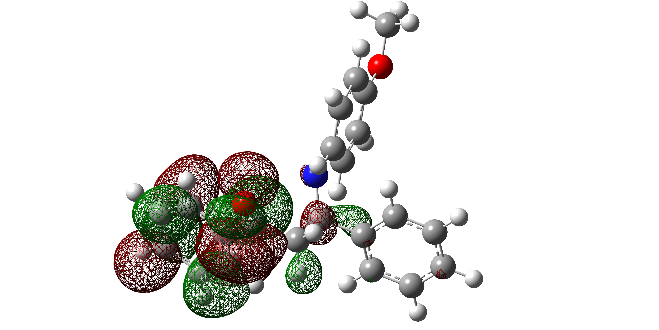 |
| --- | --- |
| HOMO-2 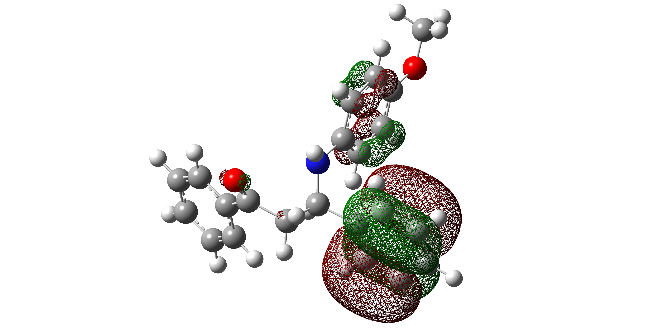 | LUMO+1 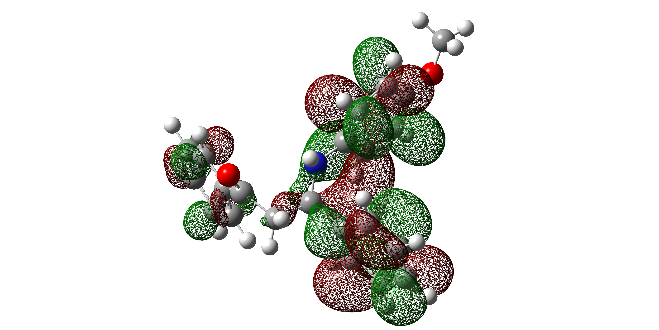 |
| HOMO-3 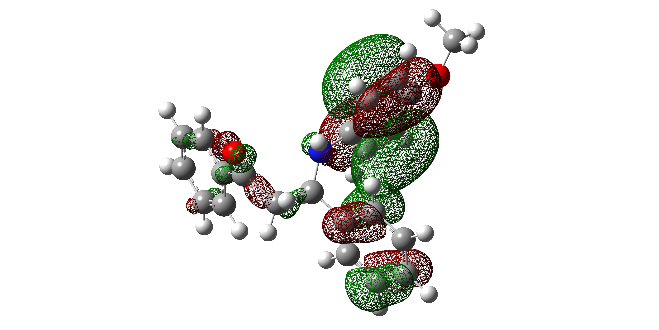 | LUMO+2 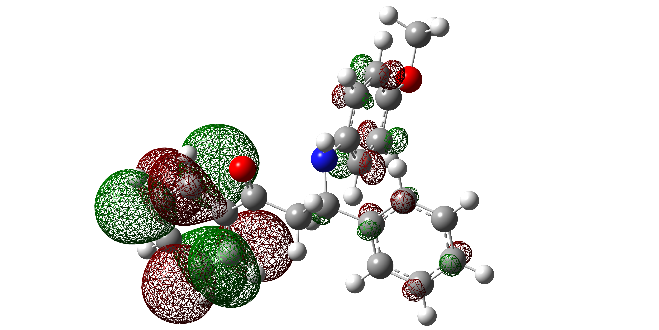 |
| HOMO-4 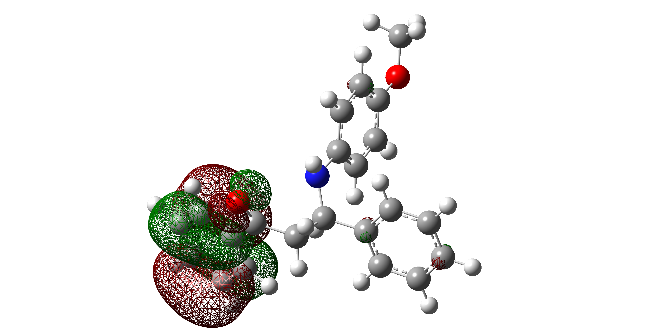 | LUMO+3 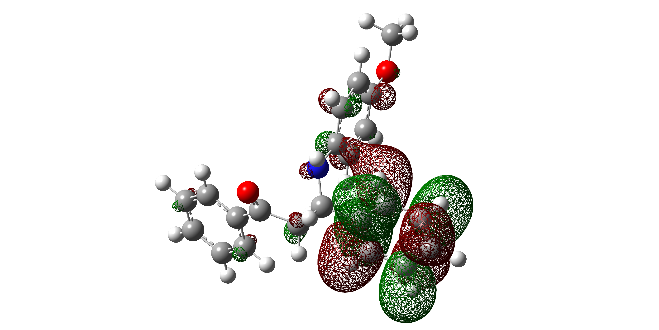 |
| HOMO-5 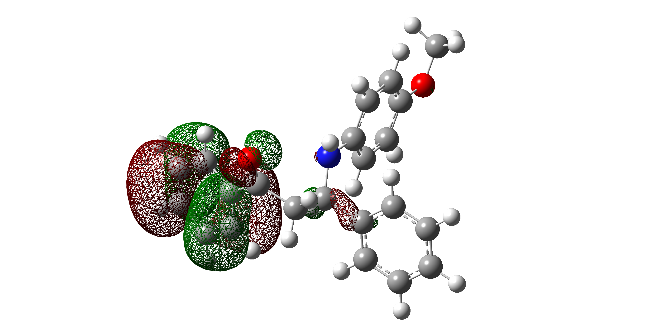 |  |

# **Figure S11**. Kohn–Sham orbitals of **MB8**.

| HOMO 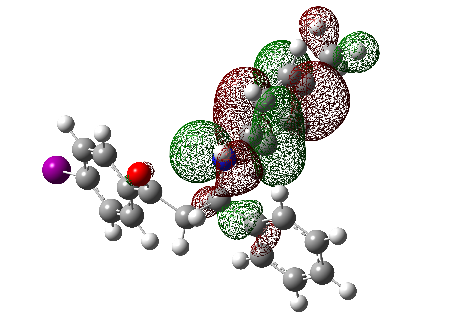 | LUMO 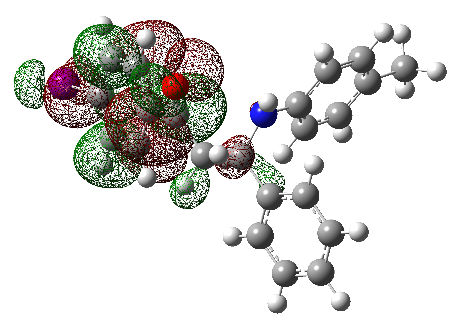 |
| --- | --- |
| HOMO-2 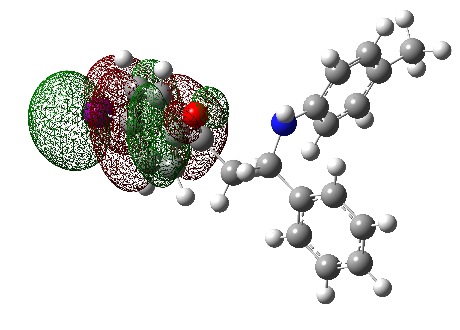 | LUMO+2 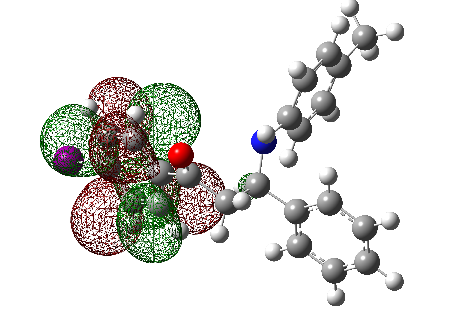 |
| HOMO-3 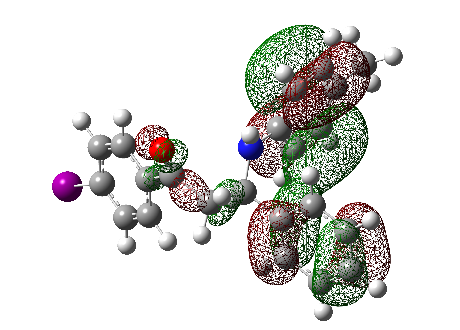 | LUMO+3 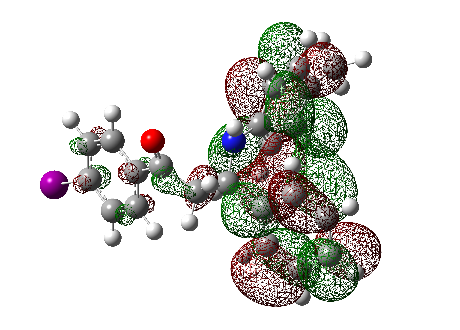 |
| HOMO-4 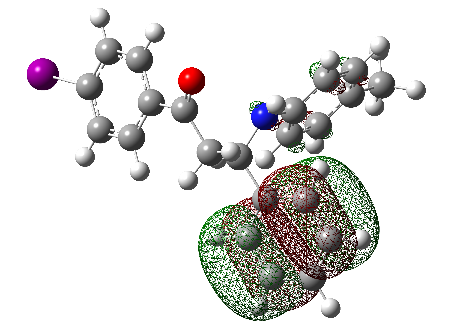 | LUMO+4 |
| HOMO-5 | LUMO+5 |
|  | LUMO+7 |

# **Figure S12**. Kohn–Sham orbitals of **MB9**.

| HOMO | LUMO |
| --- | --- |
| HOMO-2 | LUMO+2 |
| HOMO-3 | LUMO+3 |
| HOMO-4 | LUMO+4 |
| HOMO-5 | LUMO+5 |
| HOMO-7 | LUMO+8 |
| HOMO-8 | LUMO+9 |
|  | LUMO+14 |

# **Figure S13**. Kohn–Sham orbitals of **MB10**.

# **Table S1.** Electron transitions responsible for the appearance of bands in UV-Vis spectra of compounds **MB1**-**MB5.**

| **MB-1** | | | **MB-2** | | | **MB-3** | | | **MB-4** | | | **MB-5** | | |
| --- | --- | --- | --- | --- | --- | --- | --- | --- | --- | --- | --- | --- | --- | --- |
| λ(nm) | transition | | λ(nm) | transition | | λ(nm) | transition | | λ(nm) | transition | | λ(nm) | transition | |
| 203.8 | HOMO-5 | LUMO+1 | 202.6 | HOMO-5 | LUMO+2 | 202.3 | HOMO-1 | LUMO | 202.8 | HOMO-5 | LUMO+2 | 202.8 | HOMO-4 | LUMO+2 |
| 205.7 | HOMO-4 | LUMO+2 | 205.7 | HOMO-8 | LUMO |  | HOMO | LUMO+8 |  | HOMO-4 | LUMO+1 | 204.6 | HOMO-5 | LUMO |
|  | HOMO-4 | LUMO+4 |  | HOMO-5 | LUMO+2 | 202.8 | HOMO-5 | LUMO+2 | 205.7 | HOMO-4 | LUMO+2 |  | HOMO | LUMO+7 |
|  | HOMO-3 | LUMO+3 |  | HOMO-3 | LUMO+3 | 205.1 | HOMO-4 | LUMO+3 |  | HOMO-3 | LUMO+3 | 206.5 | HOMO-5 | LUMO |
| 254.5 | HOMO | LUMO+4 |  | HOMO-1 | LUMO+3 |  | HOMO-4 | LUMO+5 |  | HOMO-2 | LUMO+1 |  | HOMO-5 | LUMO+1 |
|  | HOMO | LUMO+5 | 206.1 | HOMO-8 | LUMO | 246.0 | HOMO | LUMO+4 | 247.1 | HOMO-5 | LUMO |  | HOMO-3 | LUMO+2 |
| 270.2 | HOMO-2 | LUMO |  | HOMO-5 | LUMO+2 | 265.3 | HOMO-2 | LUMO |  | HOMO-4 | LUMO |  | HOMO-1 | LUMO+1 |
| 317.8 | HOMO | LUMO | 218.8 | HOMO-2 | LUMO+2 | 308.2 | HOMO | LUMO |  | HOMO | LUMO+2 | 248.6 | HOMO-4 | LUMO |
|  |  |  | 248.2 | HOMO | LUMO+4 |  |  |  |  | HOMO | LUMO+3 |  | HOMO-3 | LUMO |
|  |  |  |  | HOMO | LUMO+7 |  |  |  |  | HOMO | LUMO+4 | 254.0 | HOMO | LUMO+3 |
|  |  |  | 265.3 | HOMO-2 | LUMO |  |  |  | 250.4 | HOMO-5 | LUMO |  | HOMO | LUMO+5 |
|  |  |  | 311.0 | HOMO | LUMO |  |  |  |  | HOMO-4 | LUMO | 299.8 | HOMO | LUMO |
|  |  |  |  |  |  |  |  |  | 300.2 | HOMO | LUMO |  |  |  |

# **Table S2.** Electron transitions responsible for the appearance of bands in UV-Vis spectra of compounds **MB6**-**MB10.**

| **MB-6** | | | **MB-7** | | | **MB-8** | | | **MB-9** | | | **MB-10** | | |
| --- | --- | --- | --- | --- | --- | --- | --- | --- | --- | --- | --- | --- | --- | --- |
| λ(nm) | transition | | λ(nm) | transition | | λ(nm) | transition | | λ(nm) | transition | | λ(nm) | transition | |
| 202.8 | HOMO-4 | LUMO+2 | 202.7 | HOMO-5 | LUMO+2 | 202.9 | HOMO-5 | LUMO+2 | 207.1 | HOMO-4 | LUMO+4 | 202.3 | HOMO-8 | LUMO |
| 203.9 | HOMO-5 | LUMO | 202.9 | HOMO-5 | LUMO+2 | 205.5 | HOMO-3 | LUMO |  | HOMO-3 | LUMO+3 |  | HOMO-7 | LUMO |
|  | HOMO-2 | LUMO+3 |  | HOMO | LUMO+8 |  | HOMO-3 | LUMO+1 |  | HOMO-3 | LUMO+5 |  | HOMO | LUMO+14 |
| 205.5 | HOMO-5 | LUMO+1 |  | HOMO | LUMO+9 |  | HOMO-2 | LUMO+3 | 218.7 | HOMO-5 | LUMO | 202.6 | HOMO-9 | LUMO |
|  | HOMO-2 | LUMO+3 | 205.9 | HOMO-5 | LUMO | 206.0 | HOMO-5 | LUMO |  | HOMO-2 | LUMO+2 |  | HOMO-3 | LUMO+4 |
|  | HOMO-1 | LUMO+1 |  | HOMO-5 | LUMO+2 |  | HOMO-5 | LUMO+2 | 251.2 | HOMO | LUMO+3 | 205.7 | HOMO-9 | LUMO |
| 206.1 | HOMO-5 | LUMO |  | HOMO-4 | LUMO+2 |  | HOMO-4 | LUMO+2 |  | HOMO | LUMO+4 |  | HOMO-5 | LUMO+2 |
|  | HOMO-4 | LUMO+2 | 207.0 | HOMO-2 | LUMO+1 | 248.1 | HOMO-5 | LUMO |  | HOMO | LUMO+7 | 206.0 | HOMO-4 | LUMO+3 |
|  | HOMO-3 | LUMO+2 |  | HOMO-2 | LUMO+4 |  | HOMO-4 | LUMO | 264.8 | HOMO-2 | LUMO | 209.3 | HOMO | LUMO+8 |
| 249.7 | HOMO-4 | LUMO |  | HOMO-1 | LUMO+1 | 295.3 | HOMO | LUMO+1 |  | HOMO | LUMO+3 |  | HOMO | LUMO+10 |
|  | HOMO-3 | LUMO |  | HOMO-1 | LUMO+4 | 317.7 | HOMO | LUMO | 281.0 | HOMO | LUMO+5 | 218.7 | HOMO-2 | LUMO+1 |
|  | HOMO | LUMO+3 | 248.3 | HOMO-5 | LUMO |  |  |  |  |  |  | 256.4 | HOMO | LUMO+4 |
| 299.3 | HOMO | LUMO |  | HOMO-4 | LUMO |  |  |  |  |  |  |  | HOMO | LUMO+5 |
|  |  |  | 252.1 | HOMO-5 | LUMO |  |  |  |  |  |  | 265.1 | HOMO-2 | LUMO |
|  |  |  |  | HOMO | LUMO+2 |  |  |  |  |  |  | 294.9 | HOMO | LUMO+3 |
|  |  |  |  | HOMO | LUMO+3 |  |  |  |  |  |  | 329.8 | HOMO | LUMO |
|  |  |  |  | HOMO | LUMO+6 |  |  |  |  |  |  |  |  |  |
|  |  |  | 308.4 | HOMO | LUMO |  |  |  |  |  |  |  |  |  |

# **Table S3.** Energies (a.u.) of orbitals of compounds **MB1**-**MB5**.

| **MB-1** | | **MB-2** | | **MB-3** | | **MB-4** | | **MB-5** | |
| --- | --- | --- | --- | --- | --- | --- | --- | --- | --- |
| LUMO+5 | 0.01507 | LUMO+7 | 0.03155 | LUMO+8 | 0.03138 | LUMO+4 | 0.02338 | LUMO+7 | 0.03573 |
| LUMO+4 | 0.01095 | LUMO+5 | 0.02286 | LUMO+5 | 0.01189 | LUMO+3 | 0.01269 | LUMO+5 | 0.02433 |
| LUMO+3 | 0.00268 | LUMO+4 | 0.01233 | LUMO+4 | 0.00283 | LUMO+2 | 0.01047 | LUMO+3 | 0.01134 |
| LUMO+2 | 0.00000 | LUMO+3 | 0.00839 | LUMO+3 | 0.00000 | LUMO+1 | 0.00825 | LUMO+2 | 0.00957 |
| LUMO | -0.01051 | LUMO+2 | -0.00137 | LUMO+2 | 0.00000 | LUMO | -0.03619 | LUMO+1 | 0.00352 |
| HOMO | -0.04839 | LUMO | -0.04338 | LUMO | -0.00674 | HOMO | -0.26726 | LUMO | -0.03682 |
| HOMO-2 | -0.31260 | HOMO | -0.26750 | HOMO | -0.04341 | HOMO-2 | -0.31947 | HOMO | -0.26666 |
| HOMO-3 | -0.31292 | HOMO-1 | -0.30908 | HOMO-1 | -0.26871 | HOMO-3 | -0.32071 | HOMO-1 | -0.31036 |
| HOMO-4 | -0.32095 | HOMO-2 | -0.31149 | HOMO-2 | -0.31100 | HOMO-4 | -0.32691 | HOMO-3 | -0.32721 |
| HOMO-5 | -0.32933 | HOMO-3 | -0.31957 | HOMO-4 | -0.32113 | HOMO-5 | -0.32915 | HOMO-4 | -0.32958 |
|  |  | HOMO-4 | -0.32120 | HOMO-5 | -0.32983 |  |  | HOMO-5 | -0.32997 |
|  |  | HOMO-5 | -0.33879 |  |  |  |  |  |  |
|  |  | HOMO-8 | -0.37535 |  |  |  |  |  |  |

# **Table S4.** Energies (a.u.) of orbitals of compounds **MB6**-**MB10**.

| **MB-6** | | **MB-7** | | **MB-8** | | **MB-9** | | **MB-10** | |
| --- | --- | --- | --- | --- | --- | --- | --- | --- | --- |
| LUMO+3 | 0.01235 | LUMO+6 | 0.04103 | LUMO+3 | 0.01342 | LUMO+7 | 0.02584 | LUMO+14 | 0.05508 |
| LUMO+2 | 0.01000 | LUMO+4 | 0.03897 | LUMO+2 | 0.01049 | LUMO+5 | 0.01274 | LUMO+10 | 0.04454 |
| LUMO+1 | 0.00334 | LUMO+3 | 0.03552 | LUMO+1 | 0.00692 | LUMO+4 | 0.00830 | LUMO+8 | 0.03568 |
| LUMO | -0.03617 | LUMO+2 | 0.03141 | LUMO | -0.03482 | LUMO+3 | 0.00000 | LUMO+5 | 0.02093 |
| HOMO | -0.26774 | LUMO+1 | 0.02385 | HOMO | -0.25283 | LUMO+2 | 0.00000 | LUMO+4 | 0.01301 |
| HOMO-1 | -0.31005 | LUMO | 0.01342 | HOMO-2 | -0.31994 | LUMO | -0.00659 | LUMO+3 | 0.00666 |
| HOMO-2 | -0.32067 | HOMO | 0.01048 | HOMO-3 | -0.32232 | HOMO | -0.04268 | LUMO+2 | 0.00000 |
| HOMO-3 | -0.32691 | HOMO-1 | 0.00839 | HOMO-4 | -0.32658 | HOMO-2 | -0.30846 | LUMO | -0.00657 |
| HOMO-4 | -0.32927 | HOMO-2 | -0.03588 | HOMO-5 | -0.32867 | HOMO-3 | -0.31132 | HOMO | -0.04285 |
| HOMO-5 | -0.32956 | HOMO-4 | -0.26068 |  |  | HOMO-4 | -0.31844 | HOMO-2 | -0.30918 |
|  |  | HOMO-5 | -0.30768 |  |  | HOMO-5 | -0.32052 | HOMO-3 | -0.31133 |
|  |  | HOMO-6 | -0.31810 |  |  |  |  | HOMO-4 | -0.32028 |
|  |  | LUMO+7 | -0.32673 |  |  |  |  | HOMO-5 | -0.32289 |
|  |  | LUMO+8 | -0.32894 |  |  |  |  | HOMO-7 | -0.34119 |
|  |  | LUMO+9 | -0.34602 |  |  |  |  | HOMO-8 | -0.34611 |
|  |  |  |  |  |  |  |  | HOMO-9 | -0.35877 |

# **Table S5.** Interaction of the examined and reference compounds with the stable radical DPPH.

| Compound | DPPH scavenging ability (%) | | | | | | IC_50_ (µM) |
| --- | --- | --- | --- | --- | --- | --- | --- |
|  | 50 µM | | 100 µM | | 150 µM | |  |
|  | 20 min | 60 min | 20 min | 60 min | 20 min | 60 min |  |
| **MB1** | 4.9±1.4 | 5.3±0.6 | 5.5±0.8 | 6.0±1.3 | 6.6±0.3 | 7.1±1.4 | – |
| **MB2** | 4.0±1.9 | 5.0±2.1 | 5.2±1.3 | 5.6±0.5 | 6.7±0.4 | 11.1±0.2 | – |
| **MB3** | 5.6±2.2 | 6.9±0.2 | 7.4±1.4 | 8.9±0.3 | 8.5±0.6 | 9.8±1.4 | – |
| **MB4** | 1.8±0.9 | 2.4±0.3 | 3.7±1.6 | 5.6±0.4 | 5.1±1.2 | 6.8±1.2 | – |
| **MB5** | 2.3±1.6 | 3.1±1.6 | 3.0±1.1 | 5.0±1.3 | 3.4±0.5 | 5.2±1.2 | – |
| **MB6** | 1.7±1.3 | 3.7±1.7 | 3.6±1.5 | 6.5±1.3 | 5.2±0.7 | 7.3±0.5 | – |
| **MB7** | 8.2±0.8 | 8.9±0.9 | 10.1±1.2 | 10.9±1.0 | 11.2±1.5 | 11.8±1.2 | – |
| **MB8** | 50.2±1.5 | 56.9±1.1 | 66.4±1.6 | 73.3±1.5 | 78.1±0.5 | 82.5±0.4 | 48.4±0.1 |
| **MB9** | 7.3±1.2 | 10.8±1.1 | 10.4±0.7 | 12.4±1.3 | 13.2±1.0 | 14.6±0.6 | – |
| **MB10** | 33.6±0.1 | 38.8±1.0 | 46.6±1.1 | 52.9±1.3 | 55.8±1.4 | 60.9±1.8 | 109.3±0.3 |
| **NDGA** | – | – | – | – | – | – | 1.7±0.1 |

# **Table S6.** Calculated thermodynamical parameters (kJ mol^-1^) of antioxidant mechanisms for **MB-8** and **MB-10**, and reaction energies (kJ mol^-1^) for the reactions of these compounds with the selected radicals in methanol based on Gibbs free energies.

|  | HAT | SET-PT | | SPLET | | HAT | SET-PT | | SPLET | |
| --- | --- | --- | --- | --- | --- | --- | --- | --- | --- | --- |
|  | **MB-8** | | | | | **MB-10** | | | | |
| Thermodynamical parameters (kJ mol^-1^) | | | | | | | | | | |
| property | BDE | IP | PDE | PA | ETE | BDE | IP | PDE | PA | ETE |
|  | 355 | 464 | 87 | 234 | 316 | 350 | 470 | 75 | 242 | 303 |
| Reaction enthalpies (kJ mol^-1^) | | | | | | | | | | |
| Radical | Δ*H*_BDE_ | ΔH_IP_ | ΔH_PDE_ | ΔH_PA_ | ΔH_ETE_ | Δ*H*_BDE_ | ΔH_IP_ | ΔH_PDE_ | ΔH_PA_ | ΔH_ETE_ |
| ^•^OCH_3_ | -71 | 89 | -160 | -12 | -59 | -76 | 95 | -171 | -4 | -72 |
| ^•^OC(CH_3_)_3_ | -78 | 85 | -163 | -15 | -63 | -83 | 91 | -174 | -7 | -76 |
| ^•^OH | -135 | 25 | -160 | -12 | -123 | -140 | 31 | -171 | -4 | -136 |
| ^•^OOH | 3 | 117 | -113 | 35 | -31 | -2 | 123 | -125 | 42 | -44 |
| ^•^OOCH_3_ | 13 | 126 | -114 | 34 | -21 | 7 | 132 | -125 | 42 | -35 |
| ^•^OO-CH=CH_2_ | 8 | 88 | -80 | 67 | -59 | 3 | 95 | -92 | 75 | -72 |
| DPPH | 32 | 44 | -12 | 136 | -104 | 27 | 50 | -23 | 144 | -117 |
| O_2_^•-^ | 84 | 352 | -268 | 115 | -31 | 79 | 358 | -280 | 123 | -44 |

# **Table S7.** Calculated thermodynamical parameters (kJ mol^-1^) of antioxidant mechanisms for **MB-10**, and reaction enthalpies (kJ mol^-1^) for the reactions of this compound with the selected radicals in methanol. M052X method and 6-311++G(d,p) basis set were used for all atoms except of iodine, where def2-TZVP basis set was used, and the conductor like solvation model (CPCM).

|  | HAT | SET-PT | | SPLET | |
| --- | --- | --- | --- | --- | --- |
|  | **MB-10** | | | | |
| Thermodynamical parameters (kJ mol^-1^) | | | | | |
| property | BDE | IP | PDE | PA | ETE |
|  | 351 | 467 | 80 | 233 | 313 |
| Reaction enthalpies (kJ mol^-1^) | | | | | |
| Radical | Δ*H*_BDE_ | ΔH_IP_ | ΔH_PDE_ | ΔH_PA_ | ΔH_ETE_ |
| ^•^OCH_3_ | -66 | 92 | -158 | -4 | -62 |
| ^•^OC(CH_3_)_3_ | -73 | 92 | -165 | -11 | -62 |
| ^•^OH | -137 | 19 | -156 | -2 | -135 |
| ^•^OOH | -8 | 81 | -89 | 65 | -73 |
| ^•^OOCH_3_ | 10 | 125 | -115 | 39 | -28 |
| ^•^OO-CH=CH_2_ | 9 | 96 | -87 | 67 | -58 |
| DPPH | 32 | 53 | -21 | 133 | -101 |
| O_2_^•-^ | 85 | 343 | -259 | 123 | -38 |

# Details for calculations of thermodynamical parameters

## Thermodynamical parameters in the absence of free radical species

Antioxidants (AOH, or in this case ANH) can inactivate free radicals through the processes of H-atom abstraction, which can occur via at least three different mechanisms: hydrogen atom transfer (HAT), single electron transfer followed by proton transfer (SET-PT) and sequential proton loss electron transfer (SPLET).[1]

HAT mechanism is reflected on the H atom donating abilities which is released by the homolytic cleavage of the O−H bond, Eq. (1):

A−OH → A−O^•^ + H^•^  (1)

The first step of SET-PT mechanism is characterized with the transfer of an electron from A−OH by which the radical cation (A−OH^•+^) is formed, Eq. (2). The deprotonation of A−OH^•+^, Eq. (3), is process which is occurring in the second step of this mechanism:

A−OH → A−OH^•+^ + e^−^ (2)

A−OH^•+^ → A−O^•^ + H^+^ (3)

The SPLET mechanism is described by heterolytic cleavage of O−H bond. The first step of this mechanism is deprotonation of A−OH which results in the formation of an anion (A−O^−^), Eq. (4). In the second step, an electron is transferred from A−O^−^, Eq. (5), and corresponding radical is formed.

A−OH → A−O^−^ + H^+^ (4)

A−O^−^ → A−O^•^ + e^−^ (5)

All described mechanisms have the same net result, formation of a stable radical of antioxidant.

The reaction enthalpies related to the mentioned free radical scavenging mechanisms can be calculated by the following equations: [2]

BDE = *H*(A−O^•^) + *H*(H^•^) − *H*(A−OH) (6)

IP = *H*(A−OH^•+^) + *H*(e^−^) − *H*(A−OH) (7)

PDE = *H*(A−O^•^) + *H*(H^+^) − *H*(A−OH^•+^) (8)

PA = *H*(A−O^−^) + *H*(H^+^) − *H*(A−OH) (9)

ETE = *H*(A−O^•^) + *H*(e^−^) − *H*(A−O^−^) (10)

where *H*(A−OH), *H*(A−O^•^), *H*(A−OH^•+^), *H*(A−O^−^), *H*(H^•^), *H*(e^−^) and *H*(H^+^) are the enthalpies of parent molecule, radical, radical cation, and anion of the examined compound, hydrogen atom, electron and proton, respectively.

## Thermodynamic parameters in the presence of harmful free radicals

The enthalpies of reactants, products, as well as enthalpies of reactions with selected free radicals are calculated. The reaction with the free radical (RO^•^) can occur via three above mentioned mechanisms.

In the HAT mechanism reaction is occurring following the Eq. (11):

A−OH + RO^•^ → A−O^•^ + ROH (11)

The SET-PT mechanism takes place in two steps as it is described above in Eqs. 2 and 3, but interaction with free radicals (RO^•^) is occurring following the next equations:

A−OH + RO^•^ → A−OH^•+^ + RO^−^ (12)

A−OH^•+^ + RO^−^ → A−O^•^ + ROH (13)

The SPLET mechanism between antioxidant and free radical can be presented as follows:

A−O^−^ + RO^•^ → A−O^•^ + RO^−^ (14)

A−OH + RO^−^ → A−O^−^ + ROH (15)

The reaction of examined compound with free radical is thermodynamically favourable if it is exothermic:

Δ_r_*H* = [*H*(products) – *H*(reactants)] < 0 (16)

In radical inactivation, HAT mechanism (Eq. 11) is characterized by the H-atom transfer from the compounds **1-3** to the free radical (RO^•^). Δ_r_*H*_BDE_ can be calculated using the following equation: [3]

Δ_r_*H*_BDE_ = [*H*(A−O^•^) + *H*(ROH)] – [*H*(A−OH) + *H*(RO^•^)] (17)

The SET-PT mechanism is described with Eqs. 12 and 13. The first step is determined by Δ_r_*H*_IP_ and the second one by Δ_r_*H*_PDE_, Eqs. 18 and 19, respectively:

Δ_r_*H*_IP_ = [*H*(A−OH^•+^) + *H*(RO^–^)] – [*H*(A−OH) + *H*(RO^•^)] (18)

Δ_r_*H*_PDE_ = [*H*(A−O^•^) + *H*(ROH)] – [*H*(A−OH^•+^) + *H*(RO^–^) (19)

Δ_r_*H*_PA_ and Δ_r_*H*_ETE_ are the reaction enthalpies related to the SPLET mechanism (Eqs. 14 and 15), and they are calculated using Eqs. 20 and 21, respectively:

Δ_r_*H*_PA_ = [*H*(A−O^−^) + *H*(ROH)] – [*H*(A−OH) + *H*(RO^–^)] (20)

Δ_r_*H*_ETE_ = [*H*(A−O^•^) + *H*(RO^–^)] – [*H*(A−O^−^) + *H*(RO^•^)] (21)

All mentioned free radical scavenging mechanisms have the same reactants and the same products. As a consequence, they have the same net thermodynamic balance, Eq. (22), and one can say they could be competitive and may be occurring in parallel.

Δ_r_*H*_BDE_ = Δ_r_*H*_IP_ + Δ_r_*H*_PDE_ = Δ_r_*H*_PA_ + Δ_r_*H*_ETE_ (22)

# Spectral characterization of compounds **MB4**-**MB8**

1,3-diphenyl-3-(phenylamino)propan-1-one (**MB4**): white solid; ^1^H NMR (200 MHz, CDCl_3_): δ = 3.59 – 3.32 (m, 2H), 5.00 (dd, *J* = 7.3, 5.5 Hz, 1H), 4.55 (br s, 1H), 6.55 (dd, *J* = 8.6, 1.0 Hz, 2H), 6.65 (t, *J* = 7.3 Hz, 1H), 7.08 (dd, *J* = 8.6, 7.3 Hz, 2H), 7.27 – 7.21 (m, 1H), 7.31 (t, *J* = 7.1 Hz, 2H), 7.39-7.47 (m, 4H), 7.53-7.55 (m, 1H), 7.90 (dd, *J* = 8.3, 1.4 Hz, 1H); ^13^C NMR (50 MHz, CDCl_3_): δ = 46.27, 54.88, 113.87, 117.80, 126.37, 127.31, 128.17, 128.65, 128.78, 129.07, 133.31, 136.84, 143.00, 147.02, 198.20; IR (cm^-1^): 3385, 1671, 1599, 1512, 1292, 1221; UV (λ_max_) nm: 202, 246, 284 nm; C_21_H_19_NO (FW = 301.39): C, 83.69; N, 4.65; H, 6.35%; found: C, 83.38; N, 4.67; H, 6.33%.

3-((4-Chlorophenyl)amino)-1,3-diphenylpropan-1-one (**MB5**): white solid; ^1^H NMR (200 MHz, CDCl_3_): δ = 3.45 (m, 2H), 4.62 (br s, 1H), 4.94 (t, *J* = 6.3 Hz, 1H), 6.47 (d, *J* = 8.8 Hz, 2H), 7.02 (d, *J* = 8.8 Hz, 2H), 7.63 – 7.16 (m, 8H), 7.92 – 7.83 (m, 2H); ^13^C NMR (50 MHz, CDCl_3_): δ = 46.19, 55.03, 115.02, 122.53, 126.30, 127.49, 128.17, 128.70, 128.92, 133.44, 136.76, 142.54, 145.63, 198.13; IR (cm^-1^): 3371, 1665, 1599, 1506, 1285; UV (λ_max_) nm: 202, 251, 300 nm; C_21_H_18_ClNO (FW = 335.83): C, 75.11; N, 4.17; H, 5.40 %; found: C, 75.39; N, 4.18; H, 5.42%.

3-((4-Fluorophenyl)amino)-1,3-diphenylpropan-1-one (**MB6**): white crystals; ^1^H NMR (200 MHz, CDCl_3_): δ = 3.30-3.57 (m, 2H), 4.47 (br s, 1H), 4.92 (t, *J* = 6.4 Hz, 1H), 6.55 – 6.39 (m, 2H), 6.78 (t, *J* = 8.8 Hz, 2H), 7.37 – 7.19 (m, 4H), 7.49 – 7.38 (m, 4H), 7.55 (d, *J* = 8.5 Hz, 1H), 7.91 (d, *J* = 8.5 Hz, 2H); ^13^C NMR (50 MHz, CDCl_3_): δ = 46.35, 55.61, 114.88, 115.03, 115.26, 115.27, 126.36, 127.41, 128.16, 128.68, 128.84, 133.37, 136.84, 142.88, 143.41, 153.77, 158.46; 198.22; IR (cm^-1^): 3386, 1670, 1595, 1513, 1289; UV (λ_max_) nm: 202, 243.5, 302.4 nm; C_21_H_18_FNO (FW = 319.38): C, 78.98; N, 4.39; H, 5.68 %; found: C, 78.67; N, 4.37; H, 5.66%.

1,3-diphenyl-3-(p-tolylamino)propan-1-one (**MB7**): white powder; ^1^H NMR (200 MHz, CDCl_3_): δ = 2.17 (s, 3H), 3.35-3.54 (m, 2H), 4.94-5.00 (m, 1H), 6.48 (d, *J* = 8.3 Hz, 2H), 6.89 (d, *J* = 7.9 Hz, 2H), 7.65 – 7.14 (m, 8H), 8.03-7.81 (m, 2H), ^13^C NMR (50 MHz, CDCl_3_): δ = 20.33, 46.36, 55.10, 114.01, 126.38, 126.98, 127.26, 128.18, 128.65, 128.77, 129.58, 133.33, 136.77, 143.15, 144.70, 198.30; IR (cm^-1^): 3400, 1680, 1620, 1527, 1298; UV (λ_max_) nm: 201.5, 246.5, 300 nm; C_22_H_21_NO (FW = 315.42): C, 83.78; N, 4.44; H, 6.71 %; found: C, 83.49; N, 4.46; H, 6.73%.

3-((4-methoxyphenyl)amino)-1,3-diphenylpropan-1-one (**MB8**): white powder; ^1^H NMR (200 MHz, CDCl_3_): δ = 3.44 (t, *J* = 6.2 Hz, 2H), 3.68 (s, 3H), 4.92 (dd, *J* = 7.6, 5.4 Hz, 1H), 6.58 – 6.46 (m, 2H), 6.73 – 6.63 (m, 2H), 7.62 – 7.17 (m, 8H), 7.98 – 7.85 (m, 2H), ^13^C NMR (50 MHz, CDCl_3_): δ = 46.43, 55.71, 55.80, 114.76, 115.41, 126.44, 127.28, 128.17, 128.65, 128.76, 133.31, 136.86, 141.23, 143.26, 152.44, 198.35; IR (cm^-1^): 3380, 1664, 1598, 1518, 1281, 1040; UV (λ_max_) nm: 201.5, 244.5, 307 nm; C_22_H_21_NO_2_ (FW = 331.42): C, 79.73; N, 4.23; H, 6.39 %; found: C, 79.44; N, 4.24; H, 6.37%.

# ^1^H and ^13^C NMR spectra of **MB1**

# ^1^H and ^13^C NMR spectra of **MB2**

# ^1^H and ^13^C NMR spectra of **MB3**

# ^1^H and ^13^C NMR spectra of **MB4**

# ^1^H and ^13^C NMR spectra of **MB5**

^1^H and ^13^C NMR spectra of **MB6**

^1^H and ^13^C NMR spectra of **MB7**

# ^1^H and ^13^C NMR spectra of **MB8**

# ^1^H and ^13^C NMR spectra of **MB9**

^1^H and ^13^C NMR spectra of **MB10**

#

1. J. Rimarčík, V. Lukeš, E. Klein, M. Ilčin, *J. Mol. Struct. THEOCHEM* **2010**, *952*, 25–30.

2. E. Klein, V. Lukeš, M. Ilčin, *Chem. Phys.* **2007**, *336*, 51–57.

3. J. M. Dimitrić Marković, D. Milenković, D. Amić, A. Popović-Bijelić, M. Mojović, I. A. Pašti, Z. S. Marković, *Struct. Chem.* **2014**, *25*, 1795–1804.
